# Healthy eating and all-cause mortality among Chinese aged 80 years or older

**DOI:** 10.1186/s12966-022-01280-6

**Published:** 2022-05-26

**Authors:** Lijing L. Yan, Chaoyun Li, Siyu Zou, Yaxi Li, Enying Gong, Zhengting He, Shuai Shao, Xurui Jin, Yechu Hua, John A. Gallis, Elizabeth L. Turner

**Affiliations:** 1 Global Heath Research Center, Duke Kunshan University, No. 8 Duke Avenue, Kunshan, Jiangsu Province 215316 China; 2 School of Public Health, Wuhan University, Wuhan, Hubei Province, 430072 China; 3 The George Institute for Global Health, Chaoyang District, Beijing, 100600 China; 4 Ningbo Eye Hospital (affiliated with Wenzhou Medical University), Yinzhou District, 315040 Ningbo, China; 5 School of Public Health, Peking University Health Science Center, 38 Xueyuan Road, Haidian District, Beijing, 100191 China; 6 Shanghai Mental Health Center, Shanghai Jiao Tong University School of Medicine, Shanghai, 3210 Humin Rd, Shanghai 201108 China; 7 Department of Population Medicine and Public Health, Peking Union Medical College, Beijing, China; 8 Department of Epidemiology, Bloomberg School of Public Health, Johns Hopkins University, 615 North Wolfe Street, Baltimore, MD 21205 USA; 9 Nossal Institute for Global Health, University of Melbourne, Melbourne, VIC 3004 Australia; 10 MindRank AI Ltd, Hangzhou, Zhejiang 311113 China; 11 Department of Public Health Sciences, University of Rochester, Rochester, NY 14623 USA; 12 Duke Global Health Institute, Duke University, 310 Trent Drive, Durham, NC 27710 USA; 13 Department of Biostatistics and Bioinformatics, Duke University, Hock Plaza, 2424 Erwin Road, Durham, NC 27710 USA

**Keywords:** Dietary pattern, Healthy eating index, Mortality, Oldest-old, Chinese, CLHLS

## Abstract

**Background:**

There is little evidence of the influence of dietary patterns on mortality risk among adults 80 years or older (“oldest-old”). We evaluated the association between the Simplified Healthy Eating index (SHE-index) and mortality among Chinese oldest-old.

**Methods:**

Population-based cohort study from the Chinese Longitudinal Healthy Longevity Survey (CLHLS 1998–2014, *n* = 35 927), conducted in 22 Chinese provinces, were pooled for analysis. The first seven waves of the CLHLS (1998, 2000, 2002, 2005, 2008–09, 2011–12, and 2013–2014) were utilized, with follow-up to the last wave (2018) (range 0–21 years). The SHE-index was collected in each wave, and was constructed from intake frequency of nine dietary variables, with a higher score indicating better diet quality. Cox proportional hazards model with dietary patterns as a time-varying exposure was employed to analyze the relationship between SHE-index and mortality.

**Results:**

At baseline, the median age of all participants was 92 years (25th percentile, 85 years; 75th percentile, 100 years). In multivariable models, the hazard ratios (95% confidence intervals) for SHE-index quartile 2, quartile 3 and quartile 4 versus quartile1 were 0.91 (0.88, 0.93), 0.89 (0.86, 0.92) and 0.82 (0.78, 0.85), respectively. Results were generally consistent for men and women and in a large number of sensitivity analyses.

**Conclusions:**

Healthier eating patterns were associated with a significant reduction in the risk of all-cause mortality among Chinese oldest-old, lending support to the importance of life-long adherence to healthy diet into advanced old age.

**Supplementary Information:**

The online version contains supplementary material available at 10.1186/s12966-022-01280-6.

## Key Messages

- Dietary pattern is a holistic approach to better reflect the complexity of human diet and our eating behaviours.
- Equipped with seven waves of the China Longitudinal Health Longevity Study from 1998 to 2014, we evaluated the relationship between dietary patterns and all-cause mortality among 35 927 Chinese adults aged 80 years or older by time-varying Cox proportional hazards regression models.
- We provide potent evidence that even among adults aged 80 years or older, adherence to a healthy dietary pattern is associated with a significant and substantial reduction in overall mortality.

## Background

Adults who are 80 years or older (the “oldest-old”) constitute the fastest growing segment of populations worldwide. In China, the number of the oldest-old was approximately 19.3 million in 2010, which is estimated to climb to approximately 107 to 150 million in 2050 [1]. The average annual increase rate is more than twice that in the US and other developed countries [2]. Healthy diet is an important determinant of longevity and healthy ageing [3]. As the number of the oldest-old increases, identification of their eating habits and promotion of healthy diet among this age group will become even more important in the coming years.

For decades, the relationship between specific nutrients or single foods and disease risk or mortality has been the topic of numerous studies. A cohort study on nutrition, lifestyle and mortality in China reported that fruit and vegetable consumption were inversely, while intake of salt-preserved vegetables positively, associated with mortality risk among the oldest-old [4]. However, such an approach often fails to address the high degree of correlation among dietary components. Moreover, foods and nutrients interact or act in synergy when influencing metabolic processes [5]. Over the last three decades, scientific attention has shifted from the role of single nutrient or food to the role of dietary patterns, which is a holistic approach to better reflect the complexity of human diet and our eating behaviours [6]. The emphasis on healthy eating patterns is also a prominent new feature of the 2015–2020 Dietary Guidelines for Americans [7].

One of the most thoroughly examined dietary patterns is the Mediterranean diet, which is a model of healthy eating that brings significant nutritional and health benefits [8, 9]. In addition, there are two main approaches to identify eating patterns: knowledge-based (i.e., a priori) dietary patterns, such as the Healthy Eating Index [10], Recommended Food Score [11], and Dietary Approaches to Stop Hypertension index [12], or empirically-derived (i.e., a posteriori) dietary patterns (e.g., by using principal components analysis or k-means clustering) [13]. Many of these eating patterns have been shown to be associated with risk of non-communicable chronic diseases and mortality. The most common food groups and dietary features in prior research were fruits, vegetables, low-fat, limited salt and added sugars [4, 14]. More investigation is needed on the effects of special foods, such as tea, eggs, and beans [15].

In addition, almost all of these previous studies have been conducted among middle-aged to older adults younger than 80 years old and in high-income nations. The few studies on the oldest old had small samples (126–1283 people) [16–19] and short follow-up duration (1–5 years) [20, 21]. Since eating behaviours are influenced by socioeconomic and cultural factors, there are differences in dietary patterns between Western and Asian populations [22]. For example, a traditional Chinese diet emphasizes plant-based foods, and people usually enjoy green tea throughout the day while the Western diet emphasizes high protein and energy-dense food [23]. Studies that investigate dietary patterns in China may provide further cultural and scientific insights into the relationship between diet and mortality.

Thus, it remains unclear whether the association observed between dietary patterns and mortality holds true for the oldest-old in China where dietary traditions are quite different. Answers to this question are not only relevant to promoting the health of the oldest-old themselves but also have profound scientific, practical, and policy implications for health education and healthy ageing in general.

Based on the Chinese Longitudinal Healthy Longevity Survey (CLHLS), which is the first, largest, and longest-running longitudinal survey on the oldest-old ever conducted in a low- and middle-income country [24, 25], we constructed a Simplified Healthy Eating index (SHE-index) and investigated the association of adherence to the SHE-index with mortality from all causes among Chinese oldest-old [26].

## Methods

### Data source

The CLHLS used a multistage, stratified cluster sampling, and recruited participants from half of the counties and cities in 22 of China’s 31 provinces in 1998, 2000, 2002, 2005, 2008–09, 2011–12, 2013–14 and 2018 among the oldest-old [27]. The survey oversampled centenarians to include all in the survey areas who agreed to participate. Vital status of the study participants was ascertained in the follow-up waves and all surviving participants were re-interviewed. Loss to follow-up due to lost contact from each survey year to the next ranged from 8.3% to 20.6%. In each follow-up wave, the deceased respondents were replaced by recruiting new participants with the same sex and roughly the same age as that of the deceased respondents. Details of the study design have been published [27, 28].

### Dietary assessment and the simplified healthy eating index

Self-reported information on dietary consumption habits was collected by trained interviewers at each wave of the CLHLS. The respondents were asked to report their current food frequency for intake of nine foods: fruits, vegetables, fish, bean products, tea, garlic, egg, sugar, and salt-preserved vegetable. Each was reported in one of three categories: ‘always or almost every day’, ‘sometimes or occasionally’ or ‘rarely or never’.

Based on previous knowledge on the relationship between these food groups and health [29–31], for two out of the 9 variables, sugar and salt-preserved vegetables, the answer of ‘always or almost every day’, ‘sometimes or occasionally’ or ‘rarely or never’ at each participant’s first survey received a score of 0, 1, or 2, respectively; for the other 7 variables, the same three responses received scores of 2, 1, or 0, respectively. Scores for the 9 variables were then summed to obtain a scale ranging from 0 to 18 with higher scores indicating higher frequency for fruits, vegetables, fish, bean products, tea, garlic, and egg, while higher scores meant lower frequencies for sugar and salt-preserved vegetables. According to previous studies, we assumed higher scores indicate healthier dietary patterns [26]. The SHE-index was further classified into quartiles (Q1: 0–8; Q2: 9–10; Q3: 11–12; and Q4: 13–18) with approximately the same number of people in each group.

### Data on mortality

Vital status and date of death were collected from officially issued death certificates whenever available, and otherwise from the next-of-kin or local residential committees who were familiar with the decedents [26]. Duration of follow-up was calculated by the time interval between the first interview date and date at death. Survivors at the wave after which they were last surveyed were considered as censored at the time of the survey.

### Covariates

Trained interviewers who administered the questionnaire on diet also collected information on the following eleven covariates in each wave: age at baseline, sex, ethnicity (Han vs minority ethnic group), marital status (currently married and living with spouse, or separated/divorced/never married/widowed), years of education (0, 1–5, or $\ge$ 6), residence (urban vs. rural areas), occupation before age 60 (manual or non-manual), living arrangement (with household members, alone, or in an institution), smoking status (never, former, or current), current physical activity (yes vs. no), and drinking status (never/former, light, or heavy). Drinking status was classified according to the type, frequency, and amount of alcohol consumed by using an algorithm from Chinese dietary guidelines [32].

### Study population

To fully utilize all the data collected, we pooled the samples from the first seven waves from 1998 to 2014, with follow-up to the 2018 wave (range of follow-up 0 to 21 years) (see supplemental Figure S1 and Table S1 for the numbers of participants enrolled, lost, and died and sample characteristics for each survey wave). We did not use participants first enrolled at the 2018 wave because there is no follow-up from the 2018 wave yet. From the 37 491 participants aged over 80 years in the pooled dataset, we excluded 1564 participants who had missing values in the main exposure variables (diet) or covariates, resulting in a final sample size of 35,927 participants aged 80 years or older at baseline (8455, 6368, 4699, 6160, 7993, 1414, and 838 participants from each wave). For those who were lost to follow-up in the study, we utilized the midpoint imputation and imputed the survival time by the mid-point of the interview interval [33, 34]. The range of follow-up was 0 to 21 years (median 2.62 years, 25^th^, 75^th^ percentiles: 1.39, 5.15 years), resulting in a total of 136 164 person-years of follow-up. 72% of the participants (*n* = 25 688) died in the follow-up period.

### Statistical analysis

Baseline characteristics were presented as median (continuous variables) or frequency distribution (categorical variables). Survival curves stratified by time-varying SHE-index and by sex were examined using the extended Kaplan–Meier method and compared using the log-rank test [35]. Multivariable adjusted hazard ratios (HRs) with 95% confidence intervals (CIs) for risk of death were estimated for each of the nine dietary variables and by quartiles of SHE-index using Cox proportional hazards models. Multivariable adjusted Cox regression models were adjusted for eleven covariates (some of which were time-varying and so were updated in each wave) listed in the section above. Model assumptions were tested using graphs based on Schoenfeld residuals. As dietary pattern could change after enrollment, we, therefore, modelled dietary pattern as time-varying exposure in time-varying Cox proportional hazards regression models to minimize the misclassification of exposure and immortal time bias. Interaction terms of sex and SHE-index scores were assessed to determine whether sex was an effect modifier on the relationships between SHE-index scores and mortality. Effect modification by sex was considered significant if the Wald test for the interaction term was statistically significant at P < 0.05. Absolute rate difference was calculated as the mortality rate per 1000 person-years of the exposed group minus that of the reference group to estimate the reduction in mortality regarding SHE-index in very old adults.

A series of additional sensitivity analyses were conducted to further explore the relationship observed: multivariable models additionally controlling for systolic blood pressure and body weight (Table S3*, n* = *35 927*) or body mass index (kg/m^2^) (Table S4*, n* = *28 008*), excluding the participants with severe diseases at baseline (Table S5*, n* = *29 443*), excluding those who died within the first year of the baseline survey (Table S6*, n* = *30 620*), assuming those lost to follow-up were either alive (Table S7*, n* = *35 927*) or dead (Table S8*, n* = *35 927*) at the mid-point between the two waves, adjusting for period effects and re-estimating the model for each wave (Table S9), multivariable models among participates aged 65–79 years old (Table S10*, n* = *9 206*), classes of dietary patterns and types of food in each class identified using the principal component analysis – PCA (Table S11), multivariate models by three dietary patterns identified by the PCA (Table S12*, n* = *35 927*).

Statistical significance was set at *P-*value < 0.05 (two-sided). All analyses were performed using SAS, version 9.4 (SAS Institute Inc., Cary, NC, USA) and independently verified with Stata version 16.1 (StataCorp, College Station, TX, USA).

## Results

Table 1 shows the baseline characteristics of 35 927 participants according to the quartile of the SHE-index. The median age of all oldest-old participants was 92 years (25^th^ percentile, 85 years; 75^th^ percentile, 100 years). More than half of participants were women (61%) and lived in rural areas (58%). Those with higher SHE-index scores were most likely to be younger, better educated, currently married, living with a spouse, and living in urban areas. Baseline characteristics of participants included in this analysis were similar to the 1564 individuals who were excluded due to missing covariates (Table S2).

**Table 1:** Baseline characteristics by quartiles of the Simplified Healthy Eating Index among 35 927 Chinese adults aged 80 years or older

**Variable – n (%)**^a^	**Simplified Healthy Eating-index Quartiles**				
	**Quartile 1 (0–8)**	**Quartile 2 (9–10)**	**Quartile 3 (11–12)**	**Quartile 4 (13–18)**	**Total**
	**(*N* = 11 020)**	**(*N* = 11 745)**	**(*N* = 8 706)**	**(*N* = 4 456)**	***N* = 35 927**
**Age – median (25**^**th**^**, 75**^**th**^**)**	93 (86, 100)	92 (85, 100)	91 (85, 100)	91 (84, 99)	92 (85, 100)
**Sex**					
Men	3519 (32%)	4413 (38%)	3696 (42%)	2291 (51%)	13,919 (39%)
Women	7501 (68%)	7332 (62%)	5010 (58%)	2165 (49%)	22,008 (61%)
**Education (years of schooling)**					
None (0)	8626 (78%)	8501 (72%)	5631 (65%)	2381 (53%)	25,139 (70%)
Primary school (1–5)	1795 (16%)	2269 (19%)	1933 (22%)	1090 (24%)	7087 (20%)
Middle school or higher (> 5)	599 (5%)	975 (8%)	1142 (13%)	985 (22%)	3701 (10%)
**Ethnicity**					
The minority	671 (6%)	910 (8%)	472 (5%)	209 (5%)	2262 (6%)
Han	10,349 (94%)	10,835 (92%)	8234 (95%)	4247 (95%)	33,665 (94%)
**Marital status**					
Currently married and living with spouse	1544 (14%)	1948 (17%)	1763 (20%)	1080 (24%)	6335 (18%)
Separated/divorced/never married/Widowed	9476 (86%)	9797 (83%)	6943 (80%)	3376 (76%)	29,592 (82%)
**Place of residence**					
Urban	3710 (34%)	4629 (39%)	4222 (48%)	2612 (59%)	15,173 (42%)
Rural	7310 (66%)	7116 (61%)	4484 (52%)	1844 (41%)	20,754 (58%)
**Occupation before age 60**					
Manual	287 (3%)	478 (4%)	633 (7%)	663 (15%)	2061 (6%)
Non-manual	10,733 (97%)	11,267 (96%)	8073 (93%)	3793 (85%)	33,866 (94%)
**Co-residence**					
With household member(s)	8723 (79%)	9643 (82%)	7408 (85%)	3875 (87%)	29,649 (83%)
Alone	1905 (17%)	1568 (13%)	943 (11%)	433 (10%)	4849 (13%)
In an institution	392 (4%)	534 (5%)	355 (4%)	148 (3%)	1429 (4%)
**Smoking status**					
Current smoker	8113 (74%)	8352 (71%)	5883 (68%)	2883 (65%)	25,231 (70%)
Former smoker	1400 (13%)	1618 (14%)	1337 (15%)	845 (19%)	5200 (14%)
Never smoked	1507 (14%)	1775 (15%)	1486 (17%)	728 (16%)	5496 (15%)
**Current physical activity**					
No	9020 (82%)	9081 (77%)	6045 (69%)	2641 (59%)	26,787 (75%)
Yes	2000 (18%)	2664 (23%)	2661 (31%)	1815 (41%)	9140 (25%)
**Drinking status**^b^					
Never/former	9221 (84%)	9545 (81%)	6923 (80%)	3547 (80%)	29,236 (81%)
Light	579 (5%)	715 (6%)	618 (7%)	370 (8%)	2282 (6%)
Heavy	1220 (11%)	1485 (13%)	1165 (13%)	539 (12%)	4409 (12%)

^a^Unless otherwise stated

^b^Light drinking < 25 g/15 g per day for male/female; Heavy drinking > 25 g/15 g per day for male/female

For the individual dietary variables, fruits, vegetables, fish, salt-preserved vegetable, tea, and garlic showed a statistically significant (*P* < 0.05) relationship with all-cause mortality, as shown by the sex-stratified analyses and the combined adjusted analyses (Table 2). In total, participants who always ate vegetables had the lowest risk of mortality (HR 0.77, 95% CI 0.75, 0.84), compared with those who rarely or never ate vegetables.

**Table 2:** Multivariable-adjusted^a^ hazard ratios (HRs), and their 95% confidence intervals (CIs) of all-cause mortality for nine individual dietary components among 35 927 Chinese adults aged 80 years or older

	**Men**		**Women**		**Total**	
	**(*N*** = **13 919)**		**(*N*** = **22 008)**		**(*N*** = **35 927)**	
	**HRs**	**95% CI**	**HRs**	**95% CI**	**HRs**	**95% CI**
**Fruit**						
Rarely or never	1 (reference)		1 (reference)		1 (reference)	
Occasionally	0.95	0.90, 0.99	0.96	0.92, 0.99	0.95	0.93, 0.98
Always	0.86	0.81, 0.90	0.91	0.88, 0.95	0.89	0.86, 0.92
**Vegetable**						
Rarely or never	1 (reference)		1 (reference)		1 (reference)	
Occasionally	0.77	0.69, 0.85	0.95	0.88, 1.03	0.88	0.83, 0.94
Always	0.71	0.65, 0.78	0.84	0.79, 0.90	0.79	0.75, 0.84
**Fish**						
Rarely or never	1 (reference)		1 (reference)		1 (reference)	
Occasionally	0.92	0.88, 0.97	0.96	0.93, 1.00	0.95	0.92, 0.98
Always	0.87	0.82, 0.93	0.93	0.89, 0.98	0.91	0.87, 0.95
**Egg**						
Rarely or never	1 (reference)		1 (reference)		1 (reference)	
Occasionally	0.91	0.86, 0.97	0.98	0.94, 1.03	0.96	0.92, 0.99
Always	0.90	0.84, 0.96	0.98	0.93, 1.03	0.95	0.91, 0.99
**Bean product**						
Rarely or never	1 (reference)		1 (reference)		1 (reference)	
Occasionally	0.89	0.84, 0.95	0.97	0.93, 1.02	0.94	0.91, 0.98
Always	0.89	0.83, 0.94	0.96	0.91, 1.01	0.93	0.90, 0.97
**Salt-preserved vegetable**						
Always	1 (reference)		1 (reference)		1 (reference)	
Occasionally	0.93	0.88, 0.97	0.96	0.93, 0.99	0.95	0.92, 0.97
Rarely or never	0.92	0.87, 0.97	0.94	0.90, 0.98	0.93	0.90, 0.96
**Sugar**						
Always	1 (reference)		1 (reference)		1 (reference)	
Occasionally	0.97	0.93, 1.02	1.00	0.96, 1.04	0.99	0.96, 1.02
Rarely or never	1.05	0.99, 1.10	1.06	1.01, 1.10	1.06	1.02, 1.09
**Tea**						
Rarely or never	1 (reference)		1 (reference)		1 (reference)	
Occasionally	0.92	0.87, 0.97	0.93	0.89, 0.97	0.93	0.90, 0.96
Always	0.88	0.84, 0.92	0.94	0.90, 0.98	0.91	0.88, 0.94
**Garlic**						
Rarely or never	1 (reference)		1 (reference)		1 (reference)	
Occasionally	0.87	0.84, 0.91	0.91	0.88, 0.95	0.90	0.88, 0.92
Always	0.85	0.80, 0.90	0.89	0.85, 0.94	0.88	0.84, 0.91

^a^Adjusted for age, sex (men or women), ethnicity (Han or the minority), marital status (currently married and living with spouse, or separated/divorced/never married/widowed), years of education (0, 1–5, or ≥ 6), residence (urban or rural areas), occupation before age 60 (manual or non-manual), co-residence (with household member, alone, or in an institution), smoking status (never smoked, former smoker or current smoker), drinking status (Never/former, light, or heavily drinking), and current physical activity (Yes or no); For men and women, adjusted for all variables except sex

**Fig. 1:**
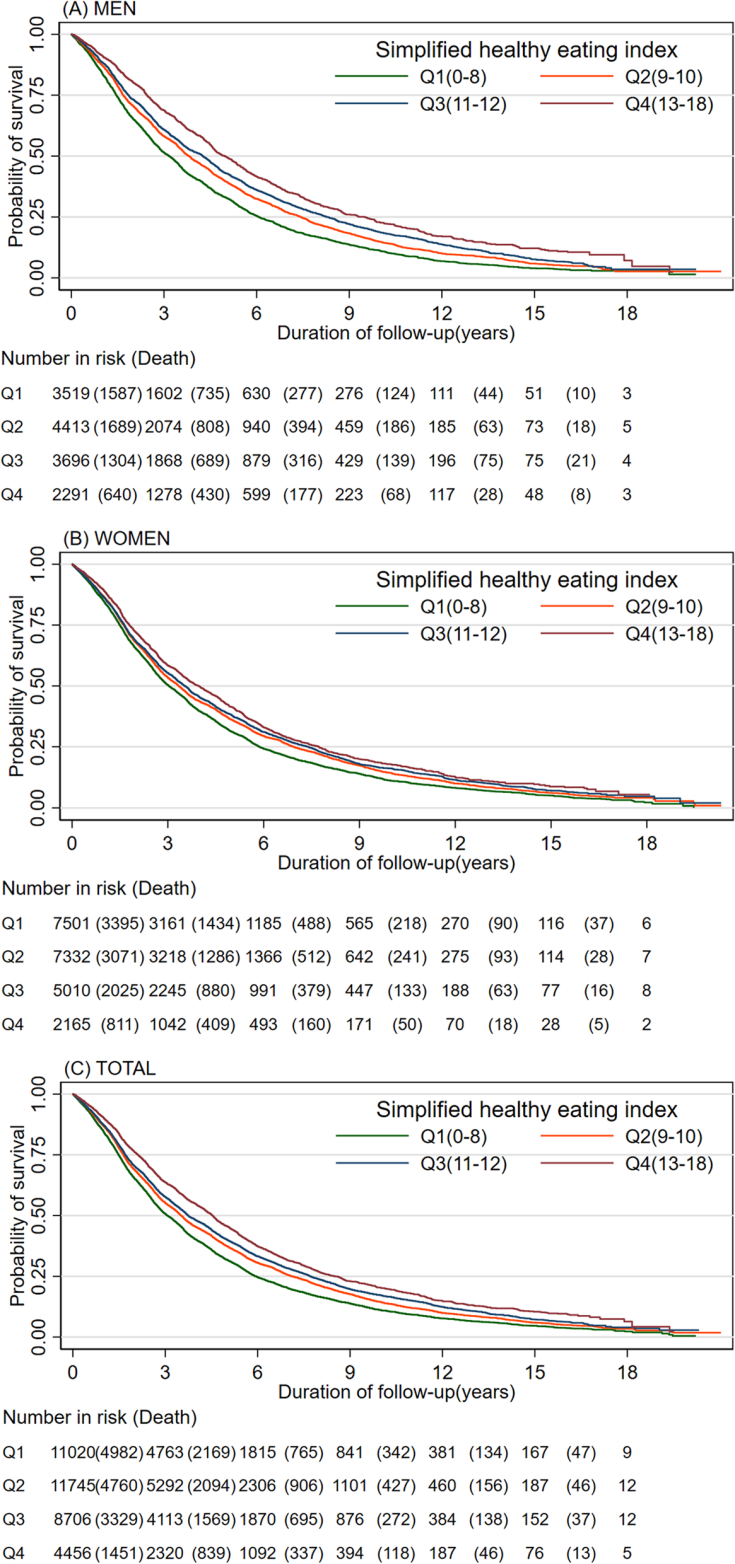
Kaplan–Meier survival curves stratified by Simplified Health Eating Index quartiles and sex for (A) Men, (B) Women and (C) Total. The tables below the graphs show the number at risk at the beginning of each time period and deaths are in parenthesis

Absolute risk differences by SHE-index quartiles are shown in Table 3. In the first quartile group with the least healthy dietary pattern, there were 71 (95% CI 81 to 61) excess deaths per 1000 person-years during the follow-up period compared to the fourth quartile with the highest healthy dietary score. Figure 1 illustrates the extended Kaplan–Meier survival curves stratified by time-varying SHE-index quartiles. The survival probability throughout the follow-up period was higher for those with higher SHE-index scores for both men and women (*P* < 0.001).

**Table 3:** Absolute risk difference and 95% confidence intervals of all-Cause mortality by quartile of SHE-index among 35 927 Chinese adults aged 80 years or older

	**Men**			**Women**			**Total**		
	**(*N*** = **13 919)**			**(*N*** = **22 008)**			**(*N*** = **35 927)**		
	**No. of Death**	**Mortality rate per 1000 Person-Years**	**Absolute Rate Difference per 1000 Person-Years (95% CI)**^a^	**No. of Death**	**Mortality rate per 1000 Person-Years**	**Absolute Rate Difference per 1000 Person-Years (95% CI)**^a^	**No. of Death**	**Mortality rate per 1000 Person-Years**	**Absolute Rate Difference per 1000 Person-Years (95% CI)**^a^
**SHE-index**									
Q1 (0–8)	1548	224	[reference]	3306	228	[reference]	4854	227	[reference]
Q2 (9–10)	1774	189	-35 (-49 to -21)	3112	209	-18 (-29 to -8)	4886	202	-25 (-34 to -17)
Q3 (11–12)	1382	172	-52 (-66 to -37)	2093	204	-23 (-35 to -12)	3475	190	-36 (-45 to -27)
Q4 (13–20)	683	134	-90 (-105 to -75)	815	179	-49 (-63 to -34)	1498	155	-71 (-81 to -61)

^a^The absolute rate difference was calculated as the mortality rate per 1000 person-years of the exposed group minus that of the reference group

**Fig. 2:**
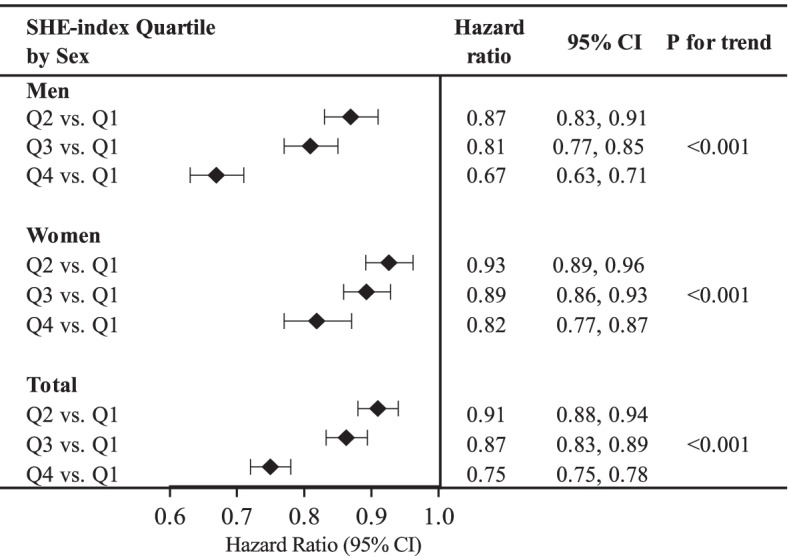
Age-adjusted Hazard Ratios stratified by sex and combined age- and sex- adjusted Hazard Ratios. Q1 represents the SHE-index score 0–8; Q2: 9–10; Q3: 11–12; Q4: 13–18. For all population, the hazard ratios were adjusted for both age and sexes; for men and women, the hazard ratios were only adjusted for age

A clear dose–response relationship emerged between the time-varying SHE-index quartiles and mortality, as shown by the age-adjusted HRs stratified by sex and by the combined age- and sex-adjusted HR (Fig. 2). Compared with the first quartile (least healthy dietary pattern), participants in the fourth quartiles had the lowest risk of mortality (HR 0.75, 95% CI 0.75, 0.78).

In multivariable Cox proportional hazard models using time-varying SHE-index score and adjusted for time-varying covariates (socio-demographic and lifestyle risk factors), the associations were slightly attenuated, but inverse relations remained among men and women, respectively, and all participants (Table 4). Compared with the first quartile, the hazard ratios (95% CI) for all participants were 0.91 (0.88, 0.93), 0.89 (0.86, 0.92) and 0.82 (0.78, 0.85) for the second, third, and fourth quartiles respectively. The effect of dietary pattern on mortality was stronger among men compared to women (p-value for interaction 0.028).

**Table 4:** Multivariable-adjusted* hazard ratios (HRs) and 95% confidence intervals (CIs) of all-cause mortality among 35 927 Chinese adults aged 80 years or older

	**Men**		**Women**		**Total**	
	**(*N* = 13 919)**		**(*N* = 22 008)**		**(*N* = 35 927)**	
	**HRs**	**95% CI**	**HRs**	**95% CI**	**HRs**	**95% CI**
**Simplified Healthy Eating-index**						
Quartile 1 (0–8)	1 (reference)		1 (reference)		1 (reference)	
Quartile 2 (9–10)	0.87	0.83–0.92	0.92	0.89–0.96	0.91	0.88–0.93
Quartile 3 (11–12)	0.85	0.80–0.89	0.92	0.88–0.96	0.89	0.86–0.92
Quartile 4 (13–18)	0.75	0.70–0.80	0.87	0.82–0.92	0.82	0.78–0.85
**Age**	1.07	1.07, 1.07	1.07	1.07, 1.07	1.07	1.07, 1.07
**Education (years of schooling)**						
None (0)	1 (reference)		1 (reference)		1 (reference)	
Primary school (1–5)	1.01	0.96–1.05	0.97	0.91–1.03	0.99	0.95–1.02
Middle school or higher (> 5)	0.95	0.89–1.01	0.85	0.76–0.96	0.92	0.87–0.97
**Ethnicity**						
Han	1 (reference)		1 (reference)		1 (reference)	
Minority	1.09	1.01–1.18	1.15	1.09–1.22	1.13	1.08–1.18
**Marital status**						
Currently married and living with spouse	1 (reference)		1 (reference)		1 (reference)	
Separated/divorced/never married/Widowed	1.32	1.26–1.39	1.46	1.34–1.58	1.36	1.30–1.41
**Place of residence**						
Urban	1 (reference)		1 (reference)		1 (reference)	
Rural	1.07	1.03–1.12	1.07	1.04–1.11	1.07	1.04–1.10
**Occupation before age 60**						
Manual	1 (reference)		1 (reference)		1 (reference)	
Non-manual	1.1	1.02–1.18	1.15	0.99–1.33	1.11	1.04–1.19
**Living arrangements**						
With household member(s)	1 (reference)		1 (reference)		1 (reference)	
Alone	0.83	0.78–0.88	0.76	0.73–0.80	0.79	0.76–0.82
In an institution	1.12	1.01–1.23	1.17	1.08–1.27	1.14	1.07–1.22
**Smoking status**						
Never smoker	1 (reference)		1 (reference)		1 (reference)	
Former smoker	1.14	1.09–1.20	1.18	1.12–1.25	1.16	1.12–1.20
Current smoker	1.05	1.00–1.10	1.02	0.96–1.10	1.04	1.00–1.09
**Physical activity**						
No	1 (reference)		1 (reference)		1 (reference)	
Yes	0.68	0.65–0.71	0.69	0.66–0.72	0.68	0.66–0.71
**Drinking status**^b^						
Never/former	1 (reference)		1 (reference)		1 (reference)	
Light	0.96	0.90–1.03	0.97	0.90–1.06	0.97	0.92–1.02
Heavy	0.91	0.86–0.96	0.97	0.92–1.03	0.94	0.90–0.98

^a^For all populations, hazard ratios were adjusted for variables listed in the table; for men and women, adjusted for all variables except sex

^b^Light drinking < 25 g/15 g per day for male/female; Heavy drinking > 25 g/15 g per day for male/female

A series of sensitivity analyses were conducted to further examine the relationship between the SHE-index and all-cause mortality. In all of these analyses except a few wave-specific models (with small sample sizes), the relationship between the SHE-index quartiles and mortality remained significant and consistent (Table S3-S12).

## Discussion

Equipped with seven waves of the China Longitudinal Health Longevity Study from 1998 to 2014, we evaluated the relationship between a simple healthy eating index and all-cause mortality among 30 771 Chinese adults aged 80 years or older at baseline (age range 80 to 124, median 92) with a total of 127 476 person-years of follow-up. The SHE-index was based on frequent intakes of fruits, vegetables, fish, eggs, bean products, garlic, and tea, and infrequent intake of sugar and salt-preserved vegetables, with higher scores indicating healthier dietary patterns. The main finding of this large prospective population-based study was that the SHE-index was inversely associated with all-cause mortality among Chinese oldest-old for both men and women. Men had a higher mortality risk than women, regardless of SHE-index. Interaction analyses showed that the effects of SHE-index were stronger in men, reflecting the greater impact of the healthy dietary pattern among men. Compared with the lowest quartile (scores between 0 and 8), the risk of dying was 9%, 11%, and 18% lower for those in the 2^nd^, 3^rd^, and 4^th^ quartiles (scores of 9–10, 11–12, and $\ge$ 13, respectively) (all *P* values < 0.001) after adjusting for socio-demographic and lifestyle risk factors. Our results provide evidence that healthy dietary patterns were associated with reduced risk of death among Chinese oldest-old.

### Comparison with other studies

In this study, the construction of the SHE-index was based on a priori knowledge of the relationships between nine specific food groups and mortality from studies primarily of middle-aged and older adults 80 years old or younger. In analyses on how *individual* food group related with mortality risk, our results among those 80 years or older were generally consistent with the previous literature [36–46]. When these individual components were included together in the SHE-index, a clear and significant relationship between dietary patterns and mortality was observed. This finding is also in line with most prior studies. For example, the Mediterranean diet is characterized by high intake of fruits, vegetables, cereals, potatoes, poultry, beans, nuts, lean fish, dairy products, small quantities of red meat, moderate alcohol consumption, and olive oil as an important fat source. Adherence to the Mediterranean diet is associated with survival not only in Greece, [47] but also in North Europe, [48] Australia, [49] Spain, [50] and the US [51]. In addition, people with higher scores of Healthy Eating Index [10, 52], Recommended Food Score [11], and Healthy Diet Indicator [53] were more likely to have lower risk of all-cause mortality.

However, nearly most of these previous studies focused on young or middle-aged adults, at most older population aged 65 and above. Using the Healthy Ageing: a Longitudinal study in Europe (HALE) data of 2,339 people in Europe and follow up for 10 years, a group examined the relationship between individual dietary, lifestyle factors and mortality, demonstrating that diet pattern along with other lifestyle factors was associated with mortality [43]. However, only a low number of deaths (905) were included in this study. Because of these low death numbers, the power of finding a significant effect of diet on mortality was largely weakened.

Using the CLHLS 1998 baseline data of 8,959 people and followed up until 2011, an Australia-based group examined the relationship between individual dietary intake variables with mortality, emphasizing the findings from fruits, vegetables, and salt [4]. Our findings extend the evidence of the beneficial effects of fruit and vegetable intake to the oldest-old.

Our present study was based on 35 927 people and up to 21 years of follow up including 25 688 deaths, with an emphasis on dietary patterns. We provide potent evidence that even among the oldest-old aged 80 and above, adherence to a healthy dietary pattern is associated with a significant and substantial reduction in overall mortality. We observed a statistically significant interaction between the effects of SHE-index and sex on mortality (men with lower SHE-index scores had higher mortality risk), and this difference between men and women was also reflected in the changes in SHE-index levels. This possible sex-specific difference should be explored in further studies.

### Public health implications

Our study suggested the additive benefits of diet on decreasing the all-cause mortality and substantiates the importance of diet on longevity in old age and adds scientific evidence for the new emphasis on healthy eating patterns in the 2015–2020 Dietary Guidelines for Americans and the 2017 Dietary Guidelines in China [7, 54].

Additionally, our study, based on a large sample of oldest-old, can refute the following two plausible perceptions of why diet may play a different and small role in older people’s health. The oldest-old are the survivors of the fittest, having been able to fend off many premature mortality risks. Thus, some people speculate that diet or other lifestyle factors may not matter to them as much as to younger people in further extending their life spans. Some older adults themselves may also hold this mentality and therefore become less motivated to maintain a healthy lifestyle.

### Strengths and limitations of study

Our study has a number of strengths. We used data from CLHLS to evaluate the association between diet pattern and all-cause mortality. Advantages of this China-wide survey were its great diversity in dietary patterns, its prospective nature, its large sample size, long follow-up period and measurements of many potential confounders. Through pooling data across seven survey waves, we are able to have complete baseline and follow-up information on more than 30 000 adults 80 years or older. The prospective nature of the study helps to minimize recall bias. In addition, taking into account the change of dietary patterns in the follow-up, time-varying models were used which enabled us to more precisely evaluate the association, and may eliminate the misclassification of exposure status to a large extent. Moreover, the length of follow-up in our study (range 0 to 21 years) is long enough to detect the true association between dietary habits and mortality.

One major limitation of our data is that we had no information on the amount of consumption and limited information on frequency of only nine dietary variables. No data were collected on other important components of a healthy eating pattern such as nuts, vegetable oil, lean meat, or dairy products. Therefore, we were only able to construct a “simplified” crude healthy eating index. In addition, in cases of eggs, the “more is better” assumption, though verified in this oldest-old sample typically with low intake, may or may not be true in other populations. It was included in our index as it represents one major food group and supplemental analyses excluding it from the SHE-index showed similar findings. Moreover, we excluded the category “meat” from our index as it is more controversially discussed among researchers and we could not simply identify it as healthy or unhealthy. Nevertheless, a clear dose–response association emerged between the SHE-index and all-cause mortality, suggesting the robustness of the relationship. A second limitation is that across the seven follow-up waves, the cumulative proportion of loss to follow-up was high (22% of all participants). Most of these losses were due to participants’ moving out of the survey areas. Nevertheless, we performed two sensitivity analyses that assumed all of the participants lost to follow-up were either dead or still alive at the mid-point of the two waves. Neither assumption considerably altered the relationship between dietary index and all-cause mortality in the main results. In addition, we did not attempt to investigate cause-specific mortality as causes of death are not always verified among oldest-old in China, especially in rural areas.

### Implications and conclusion

We found that poorer diet as measured by the SHE-index was associated with mortality from any causes among Chinese older adults aged 80 and above. Findings from this study provide new evidence on the importance of eating patterns for longevity even among the oldest-old. Our study dispels myths related to the reduced significance of diet among those who have reached old age. It provides motivation for young and old people alike to adopt and maintain healthy eating patterns for living a long life. Along with previous research, our study supports the recommendations to consume healthy diets throughout the life course.

## Supplementary Information

**Additional file 1.**

## Data Availability

The CLHLS data are publicly available at Peking University Open Research Data (http://opendata.pku.edu.cn/). The data used in the present study can be made available through reasonable request to PKU Opendata.

## Abbreviations

SHE: Simplified Healthy Eating
HRs: Hazard ratios
CI: Confidence interval
CLHLS: Chinese Longitudinal Healthy Longevity Survey
HALE: Healthy Ageing: a Longitudinal study in Europe
PCA: Principal components analysis
ARD: Absolute rate difference
