# Supplementary material for: Healthy eating and all-cause mortality among Chinese aged 80 years or older
Source: Int J Behav Nutr Phys Act. 2022 May 26;19:60. doi: 10.1186/s12966-022-01280-6 (PMC9137098; doi:10.1186/s12966-022-01280-6)
Supplement: Supplementary file 1 — Additional file 1. [file 12966_2022_1280_MOESM1_ESM.docx]

Supplementary Files: Results from the Series of Sensitivity Analyses

[**Figure S1. Numbers of participants enrolled, lost, and died during the follow-up period by survey year** 2](#_Toc93880257)

[**Table S1. General characteristic of the seven survey waves** 3](#_Toc93880258)

[**Table S2. Comparison of baseline characteristics between included and excluded subjects** 4](#_Toc93880259)

[**Table S3. Multivariate (including systolic blood pressure and body weight) adjusted hazard ratios and 95% confidence intervals of all-cause mortality by quartile of SHE-index among old population (n=35 927). *** 6](#_Toc93880260)

[**Table S4. Multivariate (including body mass index categories) adjusted hazard ratios and 95% confidence intervals of all-cause mortality by quartile of SHE-index among old population (n=28 008). *** 8](#_Toc93880261)

[**Table S5. Multivariate adjusted hazard ratios and 95% confidence intervals of all-cause mortality by quartile of SHE-index among old population without severe diseases (cancer, diabetes and cardiovascular diseases) (n=29 443).** 10](#_Toc93880262)

[**Table S6. Multivariate adjusted hazard ratios and 95% confidence intervals (CIs) of all-cause mortality by quartile of SHE-index among old population who survived more than 1 year (n=30 620). *** 12](#_Toc93880263)

[**Table S7. Multivariate adjusted hazard ratios and 95% confidence intervals of all-cause mortality by quartile of SHE-index among old population including lost-to follow up subjects (n=35 927). Subjects lost to follow up were assumed to be alive but lost to follow up in the middle time of surveyed wave. *** 14](#_Toc93880264)

[**Table S8 Multivariate adjusted hazard ratios and 95% confidence intervals (CIs) of all-cause mortality by quartile of SHE-index among old population including lost-to follow up subjects (n=35 927). Subjects lost to follow up were assumed to be dead but lost to follow up in the middle time of surveyed wave. *** 16](#_Toc93880265)

[**Table S9 Multivariate adjusted hazard ratios and 95% confidence intervals (CIs) of all-cause mortality by quartile of SHE-index among old population of different surveyed wave. *** 17](#_Toc93880266)

[**Table S10 Multivariate adjusted hazard ratios and 95% confidence intervals (CIs) of all-cause mortality by quartile of SHE-index among participates aged 65-79 (n=9 206). *** 18](#_Toc93880267)

[**Table S11. Classes of dietary patterns and types of food in each class identified using Principal component analysis (Promax rotated). *** 20](#_Toc93880268)

[**Table S12. Multivariate adjusted hazard ratios and 95% confidence intervals of all-cause mortality by three dietary patterns among old population (n=35 927). *** 20](#_Toc93880269)


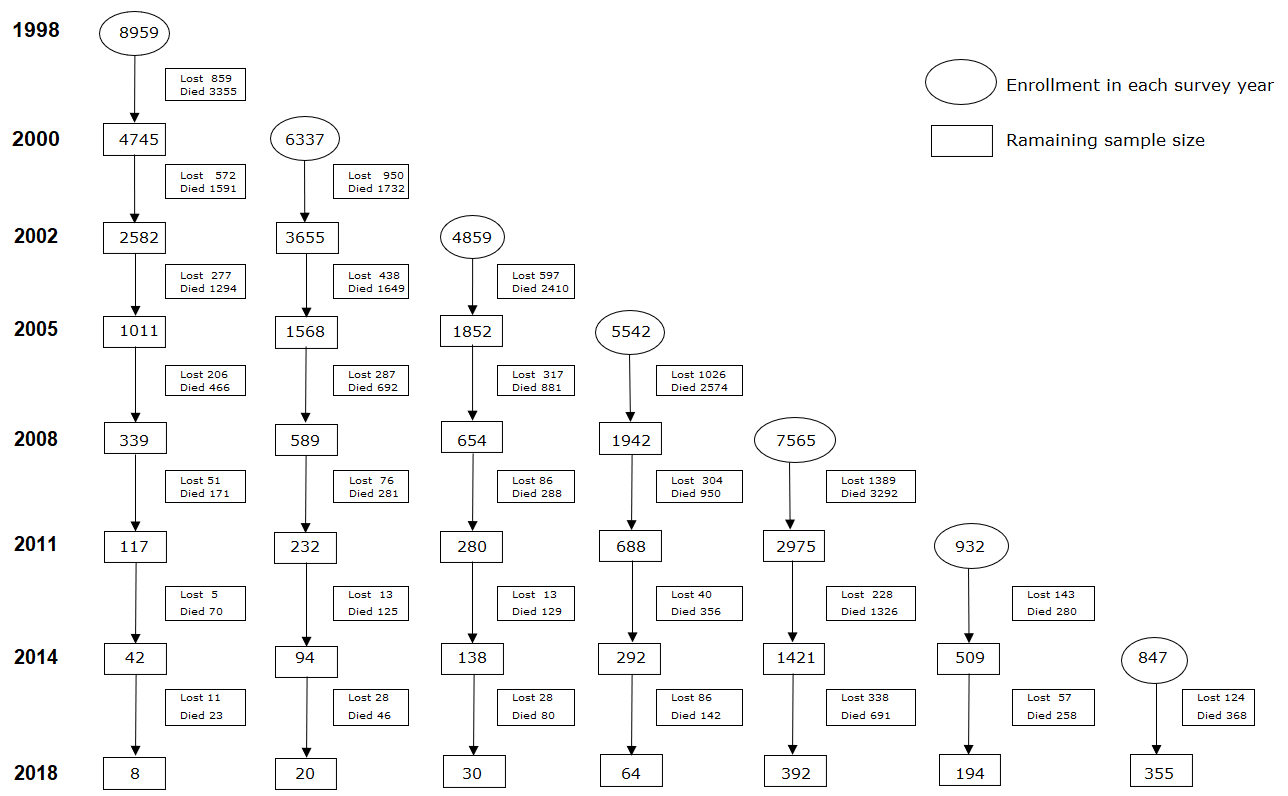


**Figure S1. Numbers of participants enrolled, lost, and died during the follow-up period by survey year**

**Table S1. General characteristic of the seven survey waves**

| **Survey Year** | **Total population^a^** | **Total oldest-old^a^** | **New Enrollment participants^b^** | **New Enrollment oldest-old^b^** | **Included** **oldest-old^c^** | **Mean age (SD)** | **Male (%)** |
| --- | --- | --- | --- | --- | --- | --- | --- |
| 1998 | 9093 | 8959 | 9093 | 8959 | 8455 | 92.4 (7.6) | 39.6 |
| 2000 | 11120 | 11082 | 6368 | 6337 | 6368 | 91.4 (7.6) | 41.8 |
| 2002 | 16064 | 11096 | 9748 | 4859 | 4699 | 92.6 (7.5) | 39.7 |
| 2005 | 15633 | 9973 | 7459 | 5542 | 6160 | 92.8 (7.2) | 39.2 |
| 2008 | 16951 | 11089 | 9479 | 7565 | 7993 | 92.7 (7.4) | 38.6 |
| 2011 | 9755 | 5224 | 1340 | 932 | 1414 | 92.2 (7.7) | 39.9 |
| 2014 | 7191 | 3343 | 1125 | 847 | 838 | 91.7 (7.4) | 40 |
| Total | 85807 | 60766 | 44612 | 35041 | 35927 | 92.1 (7.8) | 38.8 |

a Including subjects from the previous wave

b New enrollment in each survey year

c Oldest-old: Adults aged 80 years or older

**Table S2. Comparison of baseline characteristics between included and excluded subjects**

| **Variable – n (%)*** | **Included (%)** | **Excluded (%)** |
| --- | --- | --- |
| **N** | 35927 | 1564 |
| **Age – median (25^th^, 75^th^)** | 92 (85, 100) | 92 (85, 99) |
| **Gender** |  |  |
| Men | 13919 (39%) | 656 (42%) |
| Women | 22008 (61%) | 908 (58%) |
| **Education** |  |  |
| None (0 year) | 25139 (70%) | 553(35%) |
| Primary school (1-5 years) | 7087 (20%) | 151(10%) |
| Middle school or higher (>6 years) | 3701 (10%) | 80(5%) |
| Missing | 0 | 780(50%) |
| **Ethnicity** |  |  |
| The minority | 2262 (6%) | 500(32%) |
| Han | 33665 (94%) | 1,039(66%) |
| Missing | 0 | 25(2%) |
| **Marital status** |  |  |
| Currently married and living with spouse | 6335 (18%) | 363(23%) |
| Separated/divorced/never married/widowed | 29592 (82%) | 1,166(75%) |
| Missing | 0 | 35(2%) |
| **Place of residence** |  |  |
| Urban | 15173 (42%) | 673(43%) |
| Rural | 20754 (58%) | 836(53%) |
| Missing | 0 | 55(4%) |
| **Occupation before age 60** |  |  |
| Manual | 2061 (6%) | 60(4%) |
| Non-manual | 33866 (94%) | 1,424(91%) |
| Missing | 0 | 80(5%) |
| **Co-residence** |  |  |
| With household member(s) | 29649 (83%) | 1,169(75%) |
| Alone | 4849 (13%) | 275(18%) |
| In an institution | 1429 (4%) | 84(5%) |
| Missing | 0 | 36(2%) |
| **Smoking status** |  |  |
| Never smoked | 25231 (70%) | 1021(65%) |
| Former smoker | 5200 (14%) | 193(12%) |
| Current smoke | 5496 (15%) | 180(12%) |
| Missing | 0 | 170(11%) |
| **Current physical activity** |  |  |
| No | 26787 (75%) | 1057(68%) |
| Yes | 9140 (25%) | 361(23%) |
| Missing | 0 | 146(9%) |
| **Drinking status**** |  |  |
| Never/former | 29236 (81%) | 1169(75%) |
| Light | 2282 (6%) | 76(5%) |
| Heavy | 4409 (12%) | 158(10%) |
| Missing | 0 | 161(10%) |
| **SHE-index** |  |  |
| Median (range) | 10(1-18) | 9(2-17) |
| Q1 (0-8) | 11020 (100%) | 517(33%) |
| Q2 (9-10) | 11745 (100%) | 506(32%) |
| Q3 (11-12) | 8706 (100%) | 313(20%) |
| Q4 (13-20) | 4456 (100%) | 160(10%) |
| Missing | 0 | 68(4%) |

*n(%), unless otherwise stated

**Light drinking ≤25g/15g per day for male/female; Heavy drinking >25g/15g per day for male/female

**Table S3. Multivariate (including systolic blood pressure and body weight) adjusted hazard ratios and 95% confidence intervals of all-cause mortality by quartile of SHE-index among old population (n=35 927). ***

|  | **Men** | | |  | **Women** | | |  | **Total** | | |
| --- | --- | --- | --- | --- | --- | --- | --- | --- | --- | --- | --- |
|  | **(N=13 919)** | | |  | **(N=22 008)** | | |  | **(N=35 927)** | | |
|  | **HR** | **95% CI** | ***P*** |  | **HR** | **95% CI** | ***P*** |  | **HR** | **95% CI** | ***P*** |
| **SHE-index** |  |  |  |  |  |  |  |  |  |  |  |
| Q1 (0-8) | 1 (reference) | | |  | 1 (reference) | | |  | 1 (reference) | | |
| Q2 (9-10) | 0.90 | 0.86-0.95 | <0.001 |  | 0.95 | 0.91-0.98 | 0.005 |  | 0.93 | 0.90-0.96 | <0.001 |
| Q3 (11-12) | 0.89 | 0.84-0.94 | <0.001 |  | 0.94 | 0.90-0.98 | 0.004 |  | 0.92 | 0.89-0.95 | <0.001 |
| Q4 (13-18) | 0.79 | 0.74-0.85 | <0.001 |  | 0.90 | 0.85-0.96 | 0.001 |  | 0.85 | 0.81-0.89 | <0.001 |
| **Age** | 1.07 | 1.06-1.07 | <0.001 |  | 1.07 | 1.06-1.07 | 0.000 |  | 1.07 | 1.06-1.07 | <0.001 |
| **Education (years of schooling)** |  |  |  |  |  |  |  |  |  |  |  |
| None (0) | 1 (reference) | | |  | 1 (reference) | | |  | 1 (reference) | | |
| Primary school (1-5) | 1.01 | 0.96-1.05 | 0.809 |  | 0.99 | 0.93-1.05 | 0.809 |  | 1.00 | 0.96-1.03 | 0.869 |
| Middle school or higher (>6) | 0.95 | 0.89-1.01 | 0.097 |  | 0.88 | 0.78-0.99 | 0.028 |  | 0.93 | 0.88-0.98 | 0.006 |
| **Ethnicity** |  |  |  |  |  |  |  |  |  |  |  |
| Han | 1 (reference) | | |  | 1 (reference) | | |  | 1 (reference) | | |
| The minority | 1.13 | 1.04-1.23 | 0.002 |  | 1.20 | 1.13-1.27 | <0.001 |  | 1.17 | 1.12-1.23 | <0.001 |
| **Marital status** |  |  |  |  |  |  |  |  |  |  |  |
| Currently married and living with spouse | 1 (reference) | | |  | 1 (reference) | | |  | 1 (reference) | | |
| Separated/divorced/never married/widowed | 1.25 | 1.19-1.31 | <0.001 |  | 1.29 | 1.19-1.41 | <0.001 |  | 1.27 | 1.21-1.32 | <0.001 |
| **Place of residence** |  |  |  |  |  |  |  |  |  |  |  |
| Urban | 1 (reference) | | |  | 1 (reference) | | |  | 1 (reference) | | |
| Rural | 1.07 | 1.03-1.12 | 0.001 |  | 1.08 | 1.05-1.12 | <0.001 |  | 1.08 | 1.05-1.11 | <0.001 |
| **Occupation before age 60** |  |  |  |  |  |  |  |  |  |  |  |
| Manual | 1 (reference) | | |  | 1 (reference) | | |  | 1 (reference) | | |
| Non-manual | 1.07 | 0.99-1.15 | 0.090 |  | 1.18 | 1.01-1.37 | 0.039 |  | 1.09 | 1.02-1.17 | 0.009 |
| **Co-residence** |  |  |  |  |  |  |  |  |  |  |  |
| With household member(s) | 1 (reference) | | |  | 1 (reference) | | | | 1 (reference) | | |
| Alone | 0.86 | 0.81-0.92 | <0.001 |  | 0.80 | 0.76-0.84 | <0.001 |  | 0.82 | 0.79-0.85 | <0.001 |
| In an institution | 1.10 | 1.00-1.22 | 0.057 |  | 1.11 | 1.02-1.21 | 0.019 |  | 1.10 | 1.03-1.18 | 0.003 |
| **Smoking status** |  |  |  |  |  |  |  |  |  |  |  |
| Never smoked | 1 (reference) | | |  | 1 (reference) | | |  | 1 (reference) | | |
| Former smoker | 1.11 | 1.06-1.16 | <0.001 |  | 1.17 | 1.10-1.24 | <0.001 |  | 1.13 | 1.09-1.17 | <0.001 |
| Current smoke | 1.05 | 0.99-1.10 | 0.095 |  | 1.00 | 0.93-1.08 | 0.952 |  | 1.03 | 0.99-1.08 | 0.108 |
| **Physical activity** |  |  |  |  |  |  |  |  |  |  |  |
| No | 1 (reference) | | |  | 1 (reference) | | |  | 1 (reference) | | |
| Yes | 0.69 | 0.66-0.72 | <0.001 |  | 0.71 | 0.67-0.74 | <0.001 |  | 0.70 | 0.67-0.72 | <0.001 |
| **Drinking status**** |  |  |  |  |  |  |  |  |  |  |  |
| Never/former | 1 (reference) | | |  | 1 (reference) | | |  | 1 (reference) | | |
| Light | 0.96 | 0.90-1.03 | 0.299 |  | 0.97 | 0.89-1.05 | 0.461 |  | 0.97 | 0.92-1.02 | 0.229 |
| Heavy | 0.93 | 0.88-0.98 | 0.011 |  | 0.97 | 0.91-1.03 | 0.355 |  | 0.95 | 0.91-0.99 | 0.016 |
| **Systolic blood pressure** | 1.00 | 1.00-1.00 | 0.004 |  | 1.00 | 1.00-1.00 | 0.663 |  | 1.00 | 1.00-1.00 | 0.038 |
| **Body weight** | 0.99 | 0.99-0.99 | <0.001 |  | 0.99 | 0.98-0.99 | <0.001 |  | 0.99 | 0.99-0.99 | <0.001 |

* For all population, hazard ratios were adjusted for age, sex (men or women), ethnicity (Han or the minority), marital status (currently married and living with spouse, or separated/divorced/never married/widowed), years of education (0, 1-5, or $\geq$6), residence (urban or rural areas), occupation before age 60 (manual or non-manual), co-residence (with household member, alone, or in an institution), smoking status (never smoked, former smoker or current smoker), drinking status (Never/former, light, or heavily drinking), and current physical activity (Yes or no); For men and women, hazard ratios were adjusted for all these variables except for sex.

**Light drinking ≤25g/15g per day for male/female; Heavy drinking >25g/15g per day for male/female

**Table S4. Multivariate (including body mass index categories) adjusted hazard ratios and 95% confidence intervals of all-cause mortality by quartile of SHE-index among old population (n=28 008). ***

|  | **Men** | | | |  | | **Women** | | | |  | **Total** | | |
| --- | --- | --- | --- | --- | --- | --- | --- | --- | --- | --- | --- | --- | --- | --- |
|  | **(N=10 993)** | | | |  |  | **(N=17 015)** | | | |  | **(N=28 008)** | | |
|  | **HR** | **95% CI** | ***P*** |  | | **HR** | | **95% CI** | ***P*** |  | | **HR** | **95% CI** | ***P*** |
| **SHE-index** |  |  |  |  | |  | |  |  |  | |  |  |  |
| Q1 (0-8) | 1 (reference) | | | |  | | 1 (reference) | | | |  | 1 (reference) | | |
| Q2 (9-10) | 0.88 | 0.82-0.94 | <0.001 |  | | 0.97 | | 0.93-1.02 | 0.300 |  | | 0.94 | 0.90-0.98 | 0.001 |
| Q3 (11-12) | 0.87 | 0.81-0.93 | <0.001 |  | | 0.94 | | 0.89-1.00 | 0.040 |  | | 0.92 | 0.88-0.96 | <0.001 |
| Q4 (13-18) | 0.78 | 0.71-0.85 | <0.001 |  | | 0.92 | | 0.86-1.00 | 0.039 |  | | 0.86 | 0.81-0.91 | <0.001 |
| **Age** | 1.07 | 1.07-1.07 | <0.001 |  | | 1.07 | | 1.06-1.07 | <0.001 |  | | 1.07 | 1.07-1.07 | <0.001 |
| **Education (years of schooling)** |  |  |  |  | |  | |  |  |  | |  |  |  |
| None (0) | 1 (reference) | | | |  | | 1 (reference) | | | |  | 1 (reference) | | |
| Primary school (1-5) | 1.00 | 0.95-1.06 | 0.862 |  | | 1.00 | | 0.93-1.08 | 0.937 |  | | 1.00 | 0.95-1.04 | 0.899 |
| Middle school or higher (>6) | 0.97 | 0.90-1.05 | 0.476 |  | | 0.86 | | 0.74-0.99 | 0.038 |  | | 0.94 | 0.88-1.00 | 0.061 |
| **Ethnicity** |  |  |  |  | |  | |  |  |  | |  |  |  |
| Han | 1 (reference) | | | |  | | 1 (reference) | | | |  | 1 (reference) | | |
| The minority | 1.13 | 1.03-1.24 | 0.013 |  | | 1.22 | | 1.13-1.31 | <0.001 |  | | 1.19 | 1.12-1.26 | <0.001 |
| **Marital status** |  |  |  |  | |  | |  |  |  | |  |  |  |
| Currently married and living with spouse | 1 (reference) | | | |  | | 1 (reference) | | | |  | 1 (reference) | | |
| Separated/divorced/never married/widowed | 1.25 | 1.18-1.33 | <0.001 |  | | 1.35 | | 1.22-1.49 | <0.001 |  | | 1.29 | 1.22-1.35 | <0.001 |
| **Place of residence** |  |  |  |  | |  | |  |  |  | |  |  |  |
| Urban | 1 (reference) | | | |  | | 1 (reference) | | | |  | 1 (reference) | | |
| Rural | 1.13 | 1.07-1.20 | <0.001 |  | | 1.14 | | 1.10-1.19 | <0.001 |  | | 1.14 | 1.10-1.18 | <0.001 |
| **Occupation before age 60** |  |  |  |  | |  | |  |  |  | |  |  |  |
| Manual | 1 (reference) | | | |  | | 1 (reference) | | | |  | 1 (reference) | | |
| Non-manual | 1.08 | 0.98-1.18 | 0.112 |  | | 1.20 | | 0.99-1.46 | 0.067 |  | | 1.11 | 1.02-1.21 | 0.013 |
| **Co-residence** |  |  |  |  | |  | |  |  |  | |  |  |  |
| With household member(s) | 1 (reference) | | | |  | | 1 (reference) | | | | | 1 (reference) | | |
| Alone | 0.87 | 0.80-0.93 | <0.001 |  | | 0.79 | | 0.75-0.84 | <0.001 |  | | 0.82 | 0.78-0.86 | <0.001 |
| In an institution | 1.09 | 0.96-1.25 | 0.184 |  | | 1.04 | | 0.93-1.17 | 0.464 |  | | 1.06 | 0.97-1.15 | 0.184 |
| **Smoking status** |  |  |  |  | |  | |  |  |  | |  |  |  |
| Never smoked | 1 (reference) | | | |  | | 1 (reference) | | | |  | 1 (reference) | | |
| Former smoker | 1.10 | 1.03-1.16 | 0.002 |  | | 1.19 | | 1.11-1.28 | <0.001 |  | | 1.13 | 1.08-1.18 | <0.001 |
| Current smoke | 1.06 | 1.00-1.13 | 0.058 |  | | 1.03 | | 0.94-1.12 | 0.547 |  | | 1.06 | 1.01-1.11 | 0.027 |
| **Physical activity** |  |  |  |  | |  | |  |  |  | |  |  |  |
| No | 1 (reference) | | | |  | | 1 (reference) | | | |  | 1 (reference) | | |
| Yes | 0.68 | 0.65-0.73 | <0.001 |  | | 0.72 | | 0.68-0.76 | <0.001 |  | | 0.70 | 0.67-0.73 | <0.001 |
| **Drinking status**** |  |  |  |  | |  | |  |  |  | |  |  |  |
| Never/former | 1 (reference) | | | |  | | 1 (reference) | | | |  | 1 (reference) | | |
| Light | 1.00 | 0.92-1.10 | 0.915 |  | | 0.98 | | 0.89-1.09 | 0.769 |  | | 1.00 | 0.93-1.07 | 0.976 |
| Heavy | 0.92 | 0.86-0.99 | 0.024 |  | | 1.00 | | 0.93-1.08 | 0.921 |  | | 0.96 | 0.91-1.01 | 0.101 |
| **Systolic blood pressure** | 1.00 | 1.00-1.00 | 0.024 |  | | 1.00 | | 1.00-1.00 | 0.410 |  | | 1.00 | 1.00-1.00 | 0.486 |
| **BMI categories***** |  |  |  |  | |  | |  |  |  | |  |  |  |
| Underweight | 1.16 | 1.10-1.22 | <0.001 |  | | 1.19 | | 1.14-1.24 | <0.001 |  | | 1.18 | 1.14-1.22 | <0.001 |
| Normal | 1 (reference) | | | |  | | 1 (reference) | | | |  | 1 (reference) | | |
| Overweight | 0.90 | 0.82-0.98 | 0.020 |  | | 0.89 | | 0.81-0.98 | 0.020 |  | | 0.90 | 0.84-0.96 | <0.001 |
| Obese | 0.95 | 0.83-1.10 | 0.504 |  | | 1.03 | | 0.90-1.18 | 0.680 |  | | 0.99 | 0.90-1.09 | 0.842 |

* For all population, hazard ratios were adjusted for age, gender (men or women), ethnicity (Han or the minority), marital status (currently married and living with spouse, widowed, or separated/divorced/never married), years of education (0, 1-5, or ≥6), residence (urban or rural areas), occupation before age 60 (manual or non-manual), co-residence (with household member, alone, or in an institution), smoking status (never smoking, former smoking or current smoking), drinking status (never drinking, moderately drinking, or heavily drinking), and current physical activity (Yes or no); For men and women, hazard ratios were adjusted for all these variables except for gender.

**Light drinking ≤25g/15g per day for male/female; Heavy drinking >25g/15g per day for male/female

***BMI categories: Based on body mass index (BMI, kg/m^2^), participants were was categorized as underweight (<18.5kg/m^2^), normal (18.5-23.9kg/m^2^), overweight (24-27.9kg/m^2^) and obese (≥28kg/m^2^).

**Table S5. Multivariate adjusted hazard ratios and 95% confidence intervals of all-cause mortality by quartile of SHE-index among old population without severe diseases (cancer, diabetes and cardiovascular diseases) (n=29 443).**

|  | **Men** | | | |  | | **Women** | | |  | **Total** | | |
| --- | --- | --- | --- | --- | --- | --- | --- | --- | --- | --- | --- | --- | --- |
|  | **(N=11 058)** | | | |  |  | **(N=18 385)** | | |  | **(N=29 443)** | | |
|  | **HR** | **95% CI** | ***P*** |  | | **HR** | | **95% CI** | ***P*** |  | **HR** | **95% CI** | ***P*** |
| **SHE-index** |  |  |  |  | |  | |  |  |  |  |  |  |
| Q1 (0-8) | 1 (reference) | | | |  | | 1 (reference) | | |  | 1 (reference) | | |
| Q2 (9-10) | 0.89 | 0.84-0.94 | <0.001 |  | | 0.94 | | 0.91-0.98 | 0.005 |  | 0.93 | 0.90-0.96 | <0.001 |
| Q3 (11-12) | 0.88 | 0.83-0.93 | <0.001 |  | | 0.92 | | 0.88-0.97 | 0.001 |  | 0.91 | 0.88-0.94 | <0.001 |
| Q4 (13-20) | 0.78 | 0.72-0.84 | <0.001 |  | | 0.88 | | 0.82-0.93 | <0.001 |  | 0.83 | 0.79-0.88 | <0.001 |
| **Age** | 1.07 | 1.06-1.07 | <0.001 |  | | 1.07 | | 1.06-1.07 | <0.001 |  | 1.07 | 1.06-1.07 | <0.001 |
| **Education (years of schooling)** |  |  |  |  | |  | |  |  |  |  |  |  |
| None (0) | 1 (reference) | | | |  | | 1 (reference) | | |  | 1 (reference) | | |
| Primary school (1-5) | 1.00 | 0.96-1.05 | 0.892 |  | | 0.98 | | 0.91-1.04 | 0.470 |  | 0.99 | 0.95-1.03 | 0.635 |
| Middle school or higher (>6) | 0.94 | 0.87-1.00 | 0.062 |  | | 0.93 | | 0.81-1.06 | 0.288 |  | 0.93 | 0.87-0.98 | 0.012 |
| **Ethnicity** |  |  |  |  | |  | |  |  |  |  |  |  |
| Han | 1 (reference) | | | |  | | 1 (reference) | | |  | 1 (reference) | | |
| The minority | 1.12 | 1.03-1.22 | 0.010 |  | | 1.18 | | 1.11-1.26 | <0.001 |  | 1.16 | 1.10-1.22 | <0.001 |
| **Marital status** |  |  |  |  | |  | |  |  |  |  |  |  |
| Currently married and living with spouse | 1 (reference) | | | |  | | 1 (reference) | | |  | 1 (reference) | | |
| Separated/divorced/never married/widowed | 1.24 | 1.17-1.31 | <0.001 |  | | 1.31 | | 1.19-1.44 | <0.001 |  | 1.26 | 1.20-1.32 | <0.001 |
| **Place of residence** |  |  |  |  | |  | |  |  |  |  |  |  |
| Urban | 1 (reference) | | | |  | | 1 (reference) | | |  | 1 (reference) | | |
| Rural | 1.09 | 1.04-1.14 | <0.001 |  | | 1.08 | | 1.04-1.12 | <0.001 |  | 1.08 | 1.05-1.11 | 0.001 |
| **Occupation before age 60** |  |  |  |  | |  | |  |  |  |  |  |  |
| Manual | 1 (reference) | | | |  | | 1 (reference) | | |  | 1 (reference) | | |
| Non-manual | 1.02 | 0.93-1.12 | 0.642 |  | | 1.21 | | 1.01-1.46 | 0.042 |  | 1.07 | 0.98-1.15 | 0.122 |
| **Co-residence** |  |  |  |  | |  | |  |  |  |  |  |  |
| With household member(s) | 1 (reference) | | | |  | | 1 (reference) | | | | 1 (reference) | | |
| Alone | 0.88 | 0.82-0.94 | <0.001 |  | | 0.81 | | 0.77-0.85 | <0.001 |  | 0.83 | 0.80-0.87 | <0.001 |
| In an institution | 1.09 | 0.97-1.22 | 0.168 |  | | 1.10 | | 1.00-1.21 | 0.052 |  | 1.09 | 1.01-1.17 | 0.021 |
| **Smoking status** |  |  |  |  | |  | |  |  |  |  |  |  |
| Never smoked | 1 (reference) | | | |  | | 1 (reference) | | |  | 1 (reference) | | |
| Former smoker | 1.13 | 1.07-1.19 | <0.001 |  | | 1.12 | | 1.05-1.19 | <0.001 |  | 1.13 | 1.08-1.17 | <0.001 |
| Current smoke | 1.05 | 1.00-1.11 | 0.065 |  | | 1.04 | | 0.97-1.12 | 0.244 |  | 1.05 | 1.01-1.10 | 0.021 |
| **Physical activity** |  |  |  |  | |  | |  |  |  |  |  |  |
| No | 1 (reference) | | | |  | | 1 (reference) | | |  | 1 (reference) | | |
| Yes | 0.69 | 0.65-0.72 | <0.001 |  | | 0.71 | | 0.68-0.75 | <0.001 |  | 0.70 | 0.68-0.73 | <0.001 |
| **Drinking status**** |  |  |  |  | |  | |  |  |  |  |  |  |
| Never/former | 1 (reference) | | | |  | | 1 (reference) | | |  | 1 (reference) | | |
| Light | 0.98 | 0.91-1.06 | 0.600 |  | | 0.95 | | 0.87-1.04 | 0.269 |  | 0.97 | 0.91-1.03 | 0.268 |
| Heavy | 0.94 | 0.88-1.00 | 0.034 |  | | 0.95 | | 0.90-1.02 | 0.140 |  | 0.95 | 0.91-0.99 | 0.011 |

* For all population, hazard ratios were adjusted for age, sex (men or women), ethnicity (Han or the minority), marital status (currently married and living with spouse, or separated/divorced/never married/widowed), years of education (0, 1-5, or $\geq$6), residence (urban or rural areas), occupation before age 60 (manual or non-manual), co-residence (with household member, alone, or in an institution), smoking status (never smoked, former smoker or current smoker), drinking status (Never/former, light, or heavily drinking), and current physical activity (Yes or no); For men and women, hazard ratios were adjusted for all these variables except for sex.

**Light drinking ≤25g/15g per day for male/female; Heavy drinking >25g/15g per day for male/female

**Table S6. Multivariate adjusted hazard ratios and 95% confidence intervals (CIs) of all-cause mortality by quartile of SHE-index among old population who survived more than 1 year (n=30 620). ***

|  | **Men** | | |  | **Women** | | |  | **Total** | | |
| --- | --- | --- | --- | --- | --- | --- | --- | --- | --- | --- | --- |
|  | **(N=11 977)** | | |  | **(N=18 643)** | | |  | **(N=30 620)** | | |
|  | **HR** | **95% CI** | ***P*** |  | **HR** | **95% CI** | ***P*** |  | **HR** | **95% CI** | ***P*** |
| **SHE-index** |  |  |  |  |  |  |  |  |  |  |  |
| Q1 (0-8) | 1 (reference) | | |  | 1 (reference) | | |  | 1 (reference) | | |
| Q2 (9-10) | 0.90 | 0.85-0.95 | <0.001 |  | 0.94 | 0.90-0.98 | 0.007 |  | 0.93 | 0.90-0.96 | <0.001 |
| Q3 (11-12) | 0.87 | 0.82-0.93 | <0.001 |  | 0.91 | 0.87-0.96 | <0.001 |  | 0.90 | 0.87-0.94 | <0.001 |
| Q4 (13-20) | 0.78 | 0.72-0.84 | <0.001 |  | 0.89 | 0.84-0.95 | 0.001 |  | 0.84 | 0.80-0.88 | <0.001 |
| **Age** | 1.07 | 1.07-1.07 | <0.001 |  | 1.07 | 1.07-1.07 | <0.001 |  | 1.07 | 1.07-1.07 | <0.001 |
| **Education (years of schooling)** |  |  |  |  |  |  |  |  |  |  |  |
| None (0) | 1 (reference) | | |  | 1 (reference) | | |  | 1 (reference) | | |
| Primary school (1-5) | 1.00 | 0.96-1.05 | 0.864 |  | 0.97 | 0.91-1.04 | 0.363 |  | 0.99 | 0.95-1.02 | 0.467 |
| Middle school or higher (>6) | 0.94 | 0.88-1.01 | 0.078 |  | 0.83 | 0.73-0.94 | 0.003 |  | 0.91 | 0.86-0.96 | 0.001 |
| **Ethnicity** |  |  |  |  |  |  |  |  |  |  |  |
| Han | 1 (reference) | | |  | 1 (reference) | | |  | 1 (reference) | | |
| The minority | 1.14 | 1.05-1.24 | 0.003 |  | 1.17 | 1.09-1.25 | <0.001 |  | 1.16 | 1.10-1.22 | <0.001 |
| **Marital status** |  |  |  |  |  |  |  |  |  |  |  |
| Currently married and living with spouse | 1 (reference) | | |  | 1 (reference) | | |  | 1 (reference) | | |
| Separated/divorced/never married/widowed | 1.24 | 1.18-1.31 | <0.001 |  | 1.31 | 1.20-1.43 | <0.001 |  | 1.27 | 1.21-1.32 | <0.001 |
| **Place of residence** |  |  |  |  |  |  |  |  |  |  |  |
| Urban | 1 (reference) | | |  | 1 (reference) | | |  | 1 (reference) | | |
| Rural | 1.11 | 1.06-1.16 | <0.001 |  | 1.12 | 1.08-1.16 | <0.001 |  | 1.12 | 1.08-1.15 | <0.001 |
| **Occupation before age 60** |  |  |  |  |  |  |  |  |  |  |  |
| Manual | 1 (reference) | | |  | 1 (reference) | | |  | 1 (reference) | | |
| Non-manual | 1.08 | 1.00-1.18 | 0.047 |  | 1.14 | 0.97-1.33 | 0.122 |  | 1.10 | 1.02-1.18 | 0.009 |
| **Co-residence** |  |  |  |  |  |  |  |  |  |  |  |
| With household member(s) | 1 (reference) | | |  | 1 (reference) | | | | 1 (reference) | | |
| Alone | 0.88 | 0.82-0.94 | <0.001 |  | 0.81 | 0.77-0.86 | <0.001 |  | 0.83 | 0.80-0.87 | <0.001 |
| In an institution | 1.11 | 0.99-1.24 | 0.069 |  | 1.08 | 0.98-1.19 | 0.108 |  | 1.09 | 1.01-1.17 | 0.022 |
| **Smoking status** |  |  |  |  |  |  |  |  |  |  |  |
| Never smoked | 1 (reference) | | |  | 1 (reference) | | |  | 1 (reference) | | |
| Former smoker | 1.10 | 1.05-1.16 | <0.001 |  | 1.11 | 1.04-1.18 | 0.001 |  | 1.10 | 1.06-1.15 | <0.001 |
| Current smoke | 1.05 | 0.99-1.11 | 0.082 |  | 1.01 | 0.94-1.09 | 0.805 |  | 1.04 | 0.99-1.08 | 0.115 |
| **Physical activity** |  |  |  |  |  |  |  |  |  |  |  |
| No | 1 (reference) | | |  | 1 (reference) | | |  | 1 (reference) | | |
| Yes | 0.70 | 0.67-0.73 | <0.001 |  | 0.70 | 0.67-0.74 | <0.001 |  | 0.70 | 0.68-0.72 | <0.001 |
| **Drinking status**** |  |  |  |  |  |  |  |  |  |  |  |
| Never/former | 1 (reference) | | |  | 1 (reference) | | |  | 1 (reference) | | |
| Light | 0.96 | 0.89-1.04 | 0.315 |  | 1.01 | 0.92-1.10 | 0.872 |  | 0.98 | 0.93-1.04 | 0.536 |
| Heavy | 0.94 | 0.89-1.00 | 0.052 |  | 0.98 | 0.91-1.04 | 0.475 |  | 0.96 | 0.92-1.00 | 0.061 |

* For all population, hazard ratios were adjusted for age, sex (men or women), ethnicity (Han or the minority), marital status (currently married and living with spouse, or separated/divorced/never married/widowed), years of education (0, 1-5, or $\geq$6), residence (urban or rural areas), occupation before age 60 (manual or non-manual), co-residence (with household member, alone, or in an institution), smoking status (never smoked, former smoker or current smoker), drinking status (Never/former, light, or heavily drinking), and current physical activity (Yes or no); For men and women, hazard ratios were adjusted for all these variables except for sex.

**Light drinking ≤25g/15g per day for male/female; Heavy drinking >25g/15g per day for male/female

**Table S7. Multivariate adjusted hazard ratios and 95% confidence intervals of all-cause mortality by quartile of SHE-index among old population including lost-to follow up subjects (n=35 927). Subjects lost to follow up were assumed to be alive but lost to follow up in the middle time of surveyed wave. ***

|  | **Men** | | |  | **Women** | | |  | **Total** | | |
| --- | --- | --- | --- | --- | --- | --- | --- | --- | --- | --- | --- |
|  | **(N=13 919)** | | |  | **(N=22 008)** | | |  | **(N=35 927)** | | |
|  | **HR** | **95% CI** | ***P*** |  | **HR** | **95% CI** | ***P*** |  | **HR** | **95% CI** | ***P*** |
| **SHE-index** |  |  |  |  |  |  |  |  |  |  |  |
| Q1 (0-8) | 1 (reference) | | |  | 1 (reference) | | |  | 1 (reference) | | |
| Q2 (9-10) | 0.87 | 0.83-0.92 | <0.001 |  | 0.92 | 0.89-0.96 | <0.001 |  | 0.91 | 0.88-0.93 | <0.001 |
| Q3 (11-12) | 0.85 | 0.80-0.89 | <0.001 |  | 0.92 | 0.88-0.96 | <0.001 |  | 0.89 | 0.86-0.92 | <0.001 |
| Q4 (13-20) | 0.75 | 0.70-0.80 | <0.001 |  | 0.87 | 0.82-0.92 | <0.001 |  | 0.82 | 0.78-0.85 | <0.001 |
| **Age** | 1.07 | 1.07-1.07 | <0.001 |  | 1.07 | 1.07-1.07 | <0.001 |  | 1.07 | 1.07-1.07 | <0.001 |
| **Education (years of schooling)** |  |  |  |  |  |  |  |  |  |  |  |
| None (0) | 1 (reference) | | |  | 1 (reference) | | |  | 1 (reference) | | |
| Primary school (1-5) | 1.01 | 0.96-1.05 | 0.823 |  | 0.97 | 0.91-1.03 | 0.330 |  | 0.99 | 0.95-1.02 | 0.492 |
| Middle school or higher (>6) | 0.95 | 0.89-1.01 | 0.106 |  | 0.85 | 0.76-0.96 | 0.006 |  | 0.92 | 0.87-0.97 | 0.002 |
| **Ethnicity** |  |  |  |  |  |  |  |  |  |  |  |
| Han | 1 (reference) | | |  | 1 (reference) | | |  | 1 (reference) | | |
| The minority | 1.09 | 1.01-1.18 | 0.035 |  | 1.15 | 1.09-1.22 | <0.001 |  | 1.13 | 1.08-1.18 | <0.001 |
| **Marital status** |  |  |  |  |  |  |  |  |  |  |  |
| Currently married and living with spouse | 1 (reference) | | |  | 1 (reference) | | |  | 1 (reference) | | |
| Separated/divorced/never married/widowed | 1.32 | 1.26-1.39 | <0.001 |  | 1.46 | 1.34-1.58 | <0.001 |  | 1.36 | 1.30-1.41 | <0.001 |
| **Place of residence** |  |  |  |  |  |  |  |  |  |  |  |
| Urban | 1 (reference) | | |  | 1 (reference) | | |  | 1 (reference) | | |
| Rural | 1.07 | 1.03-1.12 | 0.001 |  | 1.07 | 1.04-1.11 | <0.001 |  | 1.07 | 1.04-1.10 | <0.001 |
| **Occupation before age 60** |  |  |  |  |  |  |  |  |  |  |  |
| Manual | 1 (reference) | | |  | 1 (reference) | | |  | 1 (reference) | | |
| Non-manual | 1.10 | 1.02-1.18 | 0.014 |  | 1.15 | 0.99-1.33 | 0.077 |  | 1.11 | 1.04-1.19 | 0.001 |
| **Co-residence** |  |  |  |  |  |  |  |  |  |  |  |
| With household member(s) | 1 (reference) | | |  | 1 (reference) | | | | 1 (reference) | | |
| Alone | 0.83 | 0.78-0.88 | <0.001 |  | 0.76 | 0.73-0.80 | <0.001 |  | 0.79 | 0.76-0.82 | <0.001 |
| In an institution | 1.12 | 1.01-1.23 | 0.032 |  | 1.17 | 1.08-1.27 | <0.001 |  | 1.14 | 1.07-1.22 | <0.001 |
| **Smoking status** |  |  |  |  |  |  |  |  |  |  |  |
| Never smoked | 1 (reference) | | |  | 1 (reference) | | |  | 1 (reference) | | |
| Former smoker | 1.14 | 1.09-1.20 | <0.001 |  | 1.18 | 1.12-1.25 | <0.001 |  | 1.16 | 1.12-1.20 | <0.001 |
| Current smoke | 1.05 | 1.00-1.10 | 0.062 |  | 1.02 | 0.96-1.10 | 0.515 |  | 1.04 | 1.00-1.09 | 0.036 |
| **Physical activity** |  |  |  |  |  |  |  |  |  |  |  |
| No | 1 (reference) | | |  | 1 (reference) | | |  | 1 (reference) | | |
| Yes | 0.68 | 0.65-0.71 | <0.001 |  | 0.69 | 0.66-0.72 | <0.001 |  | 0.68 | 0.66-0.71 | <0.001 |
| **Drinking status**** |  |  |  |  |  |  |  |  |  |  |  |
| Never/former | 1 (reference) | | |  | 1 (reference) | | |  | 1 (reference) | | |
| Light | 0.96 | 0.90-1.03 | 0.279 |  | 0.97 | 0.90-1.06 | 0.524 |  | 0.97 | 0.92-1.02 | 0.266 |
| Heavy | 0.91 | 0.86-0.96 | <0.001 |  | 0.97 | 0.92-1.03 | 0.377 |  | 0.94 | 0.90-0.98 | 0.001 |

* For all population, hazard ratios were adjusted for age, sex (men or women), ethnicity (Han or the minority), marital status (currently married and living with spouse, or separated/divorced/never married/widowed), years of education (0, 1-5, or $\geq$6), residence (urban or rural areas), occupation before age 60 (manual or non-manual), co-residence (with household member, alone, or in an institution), smoking status (never smoked, former smoker or current smoker), drinking status (Never/former, light, or heavily drinking), and current physical activity (Yes or no); For men and women, hazard ratios were adjusted for all these variables except for sex.

**Light drinking ≤25g/15g per day for male/female; Heavy drinking >25g/15g per day for male/female

**Table S8 Multivariate adjusted hazard ratios and 95% confidence intervals (CIs) of all-cause mortality by quartile of SHE-index among old population including lost-to follow up subjects** **(n=35 927). Subjects lost to follow up were assumed to be dead but lost to follow up in the middle time of surveyed wave. ***

|  | **Men** | | |  | **Women** | | |  | **Total** | | |
| --- | --- | --- | --- | --- | --- | --- | --- | --- | --- | --- | --- |
|  | **(N=13 919)** | | |  | **(N=22 008)** | | |  | **(N=35 927)** | | |
|  | **HR** | **95% CI** | ***P*** |  | **HR** | **95% CI** | ***P*** |  | **HR** | **95% CI** | ***P*** |
| **SHE-index** |  |  |  |  |  |  |  |  |  |  |  |
| Q1 (0-8) | 1 (reference) | | |  | 1 (reference) | | |  | 1 (reference) | | |
| Q2 (9-10) | 0.90 | 0.86-0.94 | <0.001 |  | 0.96 | 0.93-1.00 | 0.027 |  | 0.94 | 0.92-0.97 | <0.001 |
| Q3 (11-12) | 0.92 | 0.88-0.97 | 0.001 |  | 0.97 | 0.94-1.01 | 0.139 |  | 0.96 | 0.93-0.98 | 0.002 |
| Q4 (13-20) | 0.90 | 0.85-0.95 | <0.001 |  | 0.96 | 0.92-1.01 | 0.130 |  | 0.94 | 0.91-0.97 | 0.001 |
| **Age** | 1.06 | 1.06-1.07 | <0.001 |  | 1.06 | 1.06-1.06 | <0.001 |  | 1.06 | 1.06-1.06 | <0.001 |
| **Education (years of schooling)** |  |  |  |  |  |  |  |  |  |  |  |
| None (0) | 1 (reference) | | |  | 1 (reference) | | |  | 1 (reference) | | |
| Primary school (1-5) | 0.99 | 0.95-1.02 | 0.467 |  | 1.02 | 0.97-1.07 | 0.383 |  | 1.00 | 0.97-1.03 | 0.994 |
| Middle school or higher (>6) | 1.01 | 0.96-1.06 | 0.679 |  | 1.06 | 0.97-1.15 | 0.183 |  | 1.02 | 0.98-1.07 | 0.264 |
| **Ethnicity** |  |  |  |  |  |  |  |  |  |  |  |
| Han | 1 (reference) | | |  | 1 (reference) | | |  | 1 (reference) | | |
| The minority | 1.20 | 1.12-1.29 | <0.001 |  | 1.34 | 1.25-1.43 | <0.001 |  | 1.28 | 1.24-1.32 | <0.001 |
| **Marital status** |  |  |  |  |  |  |  |  |  |  |  |
| Currently married and living with spouse | 1 (reference) | | |  | 1 (reference) | | |  | 1 (reference) | | |
| Separated/divorced/never married/widowed | 1.25 | 1.20-1.30 | <0.001 |  | 1.34 | 1.25-1.43 | <0.001 |  | 1.28 | 1.24-1.32 | <0.001 |
| **Place of residence** |  |  |  |  |  |  |  |  |  |  |  |
| Urban | 1 (reference) | | |  | 1 (reference) | | |  | 1 (reference) | | |
| Rural | 0.93 | 0.90-0.97 | <0.001 |  | 0.92 | 0.89-0.95 | <0.001 |  | 0.92 | 0.90-0.94 | <0.001 |
| **Occupation before age 60** |  |  |  |  |  |  |  |  |  |  |  |
| Manual | 1 (reference) | | |  | 1 (reference) | | |  | 1 (reference) | | |
| Non-manual | 0.99 | 0.93-1.05 | 0.702 |  | 0.97 | 0.87-1.08 | 0.558 |  | 1.00 | 0.95-1.05 | 0.937 |
| **Co-residence** |  |  |  |  |  |  |  |  |  |  |  |
| With household member(s) | 1 (reference) | | |  | 1 (reference) | | | | 1 (reference) | | |
| Alone | 0.90 | 0.85-0.94 | <0.001 |  | 0.84 | 0.81-0.88 | <0.001 |  | 0.86 | 0.83-0.89 | <0.001 |
| In an institution | 1.21 | 1.12-1.32 | <0.001 |  | 1.24 | 1.15-1.32 | <0.001 |  | 1.22 | 1.16-1.29 | <0.001 |
| **Smoking status** |  |  |  |  |  |  |  |  |  |  |  |
| Never smoked | 1 (reference) | | |  | 1 (reference) | | |  | 1 (reference) | | |
| Former smoker | 1.10 | 1.05-1.14 | <0.001 |  | 1.16 | 1.11-1.22 | <0.001 |  | 1.12 | 1.09-1.16 | <0.001 |
| Current smoke | 1.00 | 0.96-1.04 | 0.999 |  | 1.03 | 0.97-1.09 | 0.309 |  | 1.02 | 0.98-1.05 | 0.369 |
| **Physical activity** |  |  |  |  |  |  |  |  |  |  |  |
| No | 1 (reference) | | |  | 1 (reference) | | |  | 1 (reference) | | |
| Yes | 0.75 | 0.73-0.78 | <0.001 |  | 0.75 | 0.72-0.77 | <0.001 |  | 0.75 | 0.73-0.77 | <0.001 |
| **Drinking status**** |  |  |  |  |  |  |  |  |  |  |  |
| Never/former | 1 (reference) | | |  | 1 (reference) | | |  | 1 (reference) | | |
| Light | 0.93 | 0.88-0.99 | 0.023 |  | 0.87 | 0.81-0.94 | <0.001 |  | 0.91 | 0.87-0.95 | <0.001 |
| Heavy | 0.87 | 0.83-0.91 | <0.001 |  | 0.93 | 0.88-0.98 | 0.007 |  | 0.89 | 0.86-0.93 | <0.001 |

* For all population, hazard ratios were adjusted for age, sex (men or women), ethnicity (Han or the minority), marital status (currently married and living with spouse, or separated/divorced/never married/widowed), years of education (0, 1-5, or $\geq$6), residence (urban or rural areas), occupation before age 60 (manual or non-manual), co-residence (with household member, alone, or in an institution), smoking status (never smoked, former smoker or current smoker), drinking status (Never/former, light, or heavily drinking), and current physical activity (Yes or no); For men and women, hazard ratios were adjusted for all these variables except for sex.

**Light drinking ≤25g/15g per day for male/female; Heavy drinking >25g/15g per day for male/female

**Table S9 Multivariate adjusted hazard ratios and 95% confidence intervals (CIs) of all-cause mortality by quartile of SHE-index among old population of different surveyed wave. ***

|  | **1998** | | |  | **2000** | | |  | **2002** | | |
| --- | --- | --- | --- | --- | --- | --- | --- | --- | --- | --- | --- |
|  | **(N=8 455)** | | |  | **(N=6 368)** | | |  | **(N=4 699)** | | |
|  | **HR** | **95% CI** | ***P*** |  | **HR** | **95% CI** | ***P*** |  | **HR** | **95% CI** | ***P*** |
| **SHE-index** |  |  |  |  |  |  |  |  |  |  |  |
| Q1 (0-8) | 1 (reference) | | |  | 1 (reference) | | |  | 1 (reference) | | |
| Q2 (9-10) | 0.93 | 0.86-1.01 | 0.092 |  | 0.88 | 0.82-0.96 | 0.003 |  | 0.97 | 0.91-1.04 | 0.379 |
| Q3 (11-12) | 0.93 | 0.84-1.04 | 0.196 |  | 0.89 | 0.81-0.98 | 0.021 |  | 0.93 | 0.86-1.00 | 0.041 |
| Q4 (13-20) | 0.83 | 0.71-0.97 | 0.023 |  | 0.74 | 0.64-0.86 | <0.001 |  | 0.89 | 0.81-0.97 | 0.011 |
|  | **2005** | | |  | **2008** | | |  | **2011** | | |
|  | **(N=6 160)** | | |  | **(N=7 993)** | | |  | **(N=1 414)** | | |
|  | **HR** | **95% CI** | ***P*** |  | **HR** | **95% CI** | ***P*** |  | **HR** | **95% CI** | ***P*** |
| **SHE-index** |  |  |  |  |  |  |  |  |  |  |  |
| Q1 (0-8) | 1 (reference) | | |  | 1 (reference) | | |  | 1 (reference) | | |
| Q2 (9-10) | 0.88 | 0.82-0.95 | 0.001 |  | 0.91 | 0.85-0.98 | 0.007 |  | 1.00 | 0.91-1.09 | 0.939 |
| Q3 (11-12) | 0.91 | 0.84-0.99 | 0.020 |  | 0.92 | 0.86-0.99 | 0.030 |  | 0.96 | 0.87-1.06 | 0.418 |
| Q4 (13-20) | 0.86 | 0.79-0.95 | 0.002 |  | 0.78 | 0.71-0.86 | <0.001 |  | 0.99 | 0.87-1.13 | 0.887 |
|  | **2014** | | |  | **Total** | | |  |  |  |  |
|  | **(N=838)** | | |  | **(N=35 927)** | | |  |  |  |  |
|  | **HR** | **95% CI** | ***P*** |  | **HR** | **95% CI** | ***P*** |  |  |  |  |
| **SHE-index** |  |  |  |  |  |  |  |  |  |  |  |
| Q1 (0-8) | 1 (reference) | | |  | 1 (reference) | | |  |  |  |  |
| Q2 (9-10) | 1.01 | 0.82-1.23 | 0.934 |  | 0.93 | 0.90-0.96 | <0.001 |  |  |  |  |
| Q3 (11-12) | 1.09 | 0.87-1.37 | 0.458 |  | 0.93 | 0.89-0.96 | <0.001 |  |  |  |  |
| Q4 (13-20) | 0.91 | 0.64-1.29 | 0.601 |  | 0.85 | 0.82-0.89 | <0.001 |  |  |  |  |

* For all population, hazard ratios were adjusted for age, sex (men or women), ethnicity (Han or the minority), marital status (currently married and living with spouse, or separated/divorced/never married/widowed), years of education (0, 1-5, or $\geq$6), residence (urban or rural areas), occupation before age 60 (manual or non-manual), co-residence (with household member, alone, or in an institution), smoking status (never smoked, former smoker or current smoker), drinking status (Never/former, light, or heavily drinking), and current physical activity (Yes or no); For men and women, hazard ratios were adjusted for all these variables except for sex.

**Light drinking ≤25g/15g per day for male/female; Heavy drinking >25g/15g per day for male/female.

**Table S10 Multivariate adjusted hazard ratios and 95% confidence intervals (CIs) of all-cause mortality by quartile of SHE-index among participates aged 65-79 (n=9 206). ***

|  | **Men** | | |  | **Women** | | |  | **Total** | | |
| --- | --- | --- | --- | --- | --- | --- | --- | --- | --- | --- | --- |
|  | **(N=4 329)** | | |  | **(N=4 877)** | | |  | **(N=9 206)** | | |
|  | **HR** | **95% CI** | ***P*** |  | **HR** | **95% CI** | ***P*** |  | **HR** | **95% CI** | ***P*** |
| **SHE-index** |  |  |  |  |  |  |  |  |  |  |  |
| Q1 (0-8) | 1 (reference) | | |  | 1 (reference) | | |  | 1 (reference) | | |
| Q2 (9-10) | 0.89 | 0.77-1.02 | 0.088 |  | 0.93 | 0.81-1.07 | 0.288 |  | 0.90 | 0.82-0.99 | 0.035 |
| Q3 (11-12) | 0.87 | 0.76-1.00 | 0.049 |  | 0.85 | 0.73-0.99 | 0.038 |  | 0.86 | 0.78-0.95 | 0.004 |
| Q4 (13-20) | 0.82 | 0.71-0.96 | 0.012 |  | 0.78 | 0.65-0.94 | 0.008 |  | 0.80 | 0.71-0.90 | <0.001 |
| **Age** | 1.07 | 1.06-1.08 | <0.001 |  | 1.08 | 1.06-1.09 | <0.001 |  | 1.07 | 1.07-1.08 | <0.001 |
| **Education (years of schooling)** |  |  |  |  |  |  |  |  |  |  |  |
| None (0) | 1 (reference) | | |  | 1 (reference) | | |  | 1 (reference) | | |
| Primary school (1-5) | 0.95 | 0.85-1.07 | 0.424 |  | 0.94 | 0.81-1.09 | 0.414 |  | 0.94 | 0.86-1.03 | 0.192 |
| Middle school or higher (>6) | 0.94 | 0.83-1.08 | 0.404 |  | 0.64 | 0.49-0.83 | 0.001 |  | 0.88 | 0.79-0.99 | 0.034 |
| **Ethnicity** |  |  |  |  |  |  |  |  |  |  |  |
| Han | 1 (reference) | | |  | 1 (reference) | | |  | 1 (reference) | | |
| The minority | 0.92 | 0.77-1.11 | 0.387 |  | 1.04 | 0.85-1.28 | 0.706 |  | 0.97 | 0.85-1.11 | 0.702 |
| **Marital status** |  |  |  |  |  |  |  |  |  |  |  |
| Currently married and living with spouse | 1 (reference) | | |  | 1 (reference) | | |  | 1 (reference) | | |
| Separated/divorced/never married/widowed | 1.31 | 1.16-1.47 | <0.001 |  | 1.13 | 1.00-1.28 | 0.045 |  | 1.22 | 1.12-1.33 | <0.001 |
| **Place of residence** |  |  |  |  |  |  |  |  |  |  |  |
| Urban | 1 (reference) | | |  | 1 (reference) | | |  | 1 (reference) | | |
| Rural | 1.11 | 1.00-1.24 | 0.049 |  | 1.07 | 0.95-1.21 | 0.284 |  | 1.10 | 1.01-1.19 | 0.021 |
| **Occupation before age 60** |  |  |  |  |  |  |  |  |  |  |  |
| Manual | 1 (reference) | | |  | 1 (reference) | | |  | 1 (reference) | | |
| Non-manual | 1.10 | 0.95-1.28 | 0.214 |  | 0.90 | 0.64-1.28 | 0.566 |  | 1.09 | 0.95-1.25 | 0.237 |
| **Co-residence** |  |  |  |  |  |  |  |  |  |  |  |
| With household member(s) | 1 (reference) | | |  | 1 (reference) | | |  | 1 (reference) | | |
| Alone | 0.81 | 0.68-0.97 | 0.018 |  | 0.77 | 0.65-0.90 | 0.001 |  | 0.79 | 0.71-0.89 | <0.001 |
| In an institution | 1.15 | 0.86-1.54 | 0.345 |  | 3.13 | 2.24-4.36 | <0.001 |  | 1.62 | 1.30-2.01 | <0.001 |
| **Smoking status** |  |  |  |  |  |  |  |  |  |  |  |
| Never smoked | 1 (reference) | | |  | 1 (reference) | | |  | 1 (reference) | | |
| Former smoker | 1.52 | 1.34-1.72 | <0.001 |  | 1.21 | 0.97-1.51 | 0.097 |  | 1.44 | 1.30-1.60 | <0.001 |
| Current smoke | 1.19 | 1.06-1.34 | 0.004 |  | 1.27 | 1.03-1.56 | 0.024 |  | 1.19 | 1.07-1.31 | 0.001 |
| **Physical activity** |  |  |  |  |  |  |  |  |  |  |  |
| No | 1 (reference) | | |  | 1 (reference) | | |  | 1 (reference) | | |
| Yes | 0.75 | 0.68-0.83 | <0.001 |  | 0.82 | 0.72-0.93 | 0.003 |  | 0.78 | 0.72-0.85 | <0.001 |
| **Drinking status**** |  |  |  |  |  |  |  |  |  |  |  |
| Never/former | 1 (reference) | | |  | 1 (reference) | | |  | 1 (reference) | | |
| Light | 0.77 | 0.64-0.93 | 0.007 |  | 0.69 | 0.46-1.03 | 0.069 |  | 0.75 | 0.63-0.89 | 0.001 |
| Heavy | 0.81 | 0.72-0.91 | <0.001 |  | 0.78 | 0.61-1.00 | 0.053 |  | 0.80 | 0.72-0.89 | <0.001 |

* For all population, hazard ratios were adjusted for age, gender (men or women), ethnicity (Han or the minority), marital status (currently married and living with spouse, widowed, or separated/divorced/never married), years of education (0, 1-5, or ≥6), residence (urban or rural areas), occupation before age 60 (manual or non-manual), co-residence (with household member, alone, or in an institution), smoking status (never smoking, former smoking or current smoking), drinking status (never drinking, moderately drinking, or heavily drinking), and current physical activity (Yes or no); For men and women, hazard ratios were adjusted for all these variables except for gender.

**Light drinking ≤25g/15g per day for male/female; Heavy drinking >25g/15g per day for male/female

**Table S11. Classes of dietary patterns and types of food in each class identified using Principal component analysis (Promax rotated). ***

|  | **Fresh food** | **Special food** | **Low-carbohydrate high-protein** | **Unexplained** |
| --- | --- | --- | --- | --- |
| **Fruit** | 0.5834 | 0.0196 | -0.1122 | 0.4713 |
| **Vegetable** | 0.5693 | -0.2647 | 0.1395 | 0.5127 |
| **Garlic** | -0.0166 | 0.1668 | 0.5401 | 0.5418 |
| **Fish** | 0.4478 | 0.1085 | -0.0225 | 0.6165 |
| **Egg** | 0.1409 | 0.5823 | -0.1239 | 0.396 |
| **Bean** | 0.1989 | 0.3665 | 0.0973 | 0.6145 |
| **Tea** | 0.1121 | -0.0546 | 0.4792 | 0.6651 |
| **Sugar** | -0.2746 | 0.6525 | 0.0981 | 0.4359 |
| **Salt-preserved vegetable** | -0.0446 | -0.0518 | 0.6419 | 0.4813 |

***Pattern:**

Fresh food pattern: Fruit, vegetables, and fish

Special food pattern: Garlic, tea, and little salt-preserved vegetable

Low-carbohydrate high-protein: Bean, egg, and little sugar

**Table S12. Multivariate adjusted hazard ratios and 95% confidence intervals of all-cause mortality by three dietary patterns among old population (n=35 927). ***

|  | **Men** | | |  | **Women** | | |  | **Total** | | |
| --- | --- | --- | --- | --- | --- | --- | --- | --- | --- | --- | --- |
|  | **(N=13 919)** | | |  | **(N=22 008)** | | |  | **(N=35 927)** | | |
|  | **HR** | **95% CI** | ***P*** |  | **HR** | **95% CI** | ***P*** |  | **HR** | **95% CI** | ***P*** |
| **Pattern** |  |  |  |  |  |  |  |  |  |  |  |
| P1(Fruit, vegetable, fish) | 0.92 | 0.90-0.94 | <0.001 |  | 0.94 | 0.93-0.96 | <0.001 |  | 0.93 | 0.92-0.95 | <0.001 |
| P2(Bean, egg, little sugar) | 1.01 | 0.99-1.04 | 0.198 |  | 1.02 | 1.01-1.04 | 0.003 |  | 1.02 | 1.01-1.03 | 0.001 |
| P3(Garlic, tea, low-salt vegetable) | 0.94 | 0.92-0.96 | <0.001 |  | 0.96 | 0.94-0.98 | <0.001 |  | 0.95 | 0.94-0.96 | <0.001 |
| **Age** | 1.07 | 1.06-1.07 | <0.001 |  | 1.07 | 1.07-1.07 | <0.001 |  | 1.07 | 1.07-1.07 | <0.001 |
| **Education (years of schooling)** |  |  |  |  |  |  |  |  |  |  |  |
| None (0) | 1 (reference) | | |  | 1 (reference) | | |  | 1 (reference) | | |
| Primary school (1-5) | 1.00 | 0.96-1.04 | 0.956 |  | 0.98 | 0.93-1.04 | 0.550 |  | 0.99 | 0.95-1.02 | 0.485 |
| Middle school or higher (>6) | 0.94 | 0.89-1.00 | 0.064 |  | 0.85 | 0.76-0.96 | 0.006 |  | 0.92 | 0.87-0.97 | 0.001 |
| **Ethnicity** |  |  |  |  |  |  |  |  |  |  |  |
| Han | 1 (reference) | | |  | 1 (reference) | | |  | 1 (reference) | | |
| The minority | 1.11 | 1.03-1.20 | 0.009 |  | 1.17 | 1.10-1.24 | <0.001 |  | 1.15 | 1.09-1.20 | <0.001 |
| **Marital status** |  |  |  |  |  |  |  |  |  |  |  |
| Currently married and living with spouse | 1 (reference) | | |  | 1 (reference) | | |  | 1 (reference) | | |
| Separated/divorced/never married/widowed | 1.25 | 1.19-1.31 | <0.001 |  | 1.30 | 1.20-1.41 | <0.001 |  | 1.27 | 1.22-1.32 | <0.001 |
| **Place of residence** |  |  |  |  |  |  |  |  |  |  |  |
| Urban | 1 (reference) | | |  | 1 (reference) | | |  | 1 (reference) | | |
| Rural | 1.11 | 1.06-1.15 | <0.001 |  | 1.11 | 1.07-1.14 | <0.001 |  | 1.11 | 1.08-1.13 | <0.001 |
| **Occupation before age 60** |  |  |  |  |  |  |  |  |  |  |  |
| Manual | 1 (reference) | | |  | 1 (reference) | | |  | 1 (reference) | | |
| Non-manual | 1.11 | 1.03-1.19 | 0.008 |  | 1.20 | 1.03-1.39 | 0.020 |  | 1.13 | 1.06-1.21 | <0.001 |
| **Co-residence** |  |  |  |  |  |  |  |  |  |  |  |
| With household member(s) | 1 (reference) | | |  | 1 (reference) | | |  | 1 (reference) | | |
| Alone | 0.86 | 0.81-0.91 | <0.001 |  | 0.79 | 0.76-0.84 | <0.001 |  | 0.82 | 0.79-0.85 | <0.001 |
| In an institution | 1.12 | 1.01-1.24 | 0.031 |  | 1.12 | 1.03-1.22 | 0.006 |  | 1.12 | 1.05-1.19 | 0.001 |
| **Smoking status** |  |  |  |  |  |  |  |  |  |  |  |
| Never smoked | 1 (reference) | | |  | 1 (reference) | | |  | 1 (reference) | | |
| Former smoker | 1.10 | 1.05-1.16 | <0.001 |  | 1.15 | 1.09-1.22 | <0.001 |  | 1.12 | 1.08-1.17 | <0.001 |
| Current smoke | 1.05 | 1.00-1.10 | 0.071 |  | 1.02 | 0.95-1.09 | 0.629 |  | 1.04 | 1.00-1.08 | 0.041 |
| **Physical activity** |  |  |  |  |  |  |  |  |  |  |  |
| No | 1 (reference) | | |  | 1 (reference) | | |  | 1 (reference) | | |
| Yes | 0.68 | 0.65-0.71 | <0.001 |  | 0.70 | 0.67-0.73 | <0.001 |  | 0.69 | 0.66-0.71 | <0.001 |
| **Drinking status**** |  |  |  |  |  |  |  |  |  |  |  |
| Never/former | 1 (reference) | | |  | 1 (reference) | | |  | 1 (reference) | | |
| Light | 0.98 | 0.91-1.05 | 0.567 |  | 0.99 | 0.91-1.07 | 0.760 |  | 0.98 | 0.93-1.04 | 0.561 |
| Heavy | 0.93 | 0.88-0.99 | 0.013 |  | 0.97 | 0.92-1.03 | 0.350 |  | 0.95 | 0.91-0.99 | 0.015 |

* For all population, hazard ratios were adjusted for age, gender (men or women), ethnicity (Han or the minority), marital status (currently married and living with spouse, widowed, or separated/divorced/never married), years of education (0, 1-5, or ≥6), residence (urban or rural areas), occupation before age 60 (manual or non-manual), co-residence (with household member, alone, or in an institution), smoking status (never smoking, former smoking or current smoking), drinking status (never drinking, moderately drinking, or heavily drinking), and current physical activity (Yes or no); For men and women, hazard ratios were adjusted for all these variables except for gender.

**Light drinking ≤25g/15g per day for male/female; Heavy drinking >25g/15g per day for male/female
